# Organic‒inorganic semi-interpenetrating networks with orthogonal light- and magnetic-responsiveness for smart photonic gels

**DOI:** 10.1038/s41467-023-36706-7

**Published:** 2023-02-22

**Authors:** Minghao Wang, Chen Nie, Junbang Liu, Si Wu

**Affiliations:** 1 CAS Key Laboratory of Soft Matter Chemistry, Anhui Key Laboratory of Optoelectronic Science and Technology, Department of Polymer Science and Engineering, University of Science and Technology of China, 230026 Hefei, China; 2 CAS Key Laboratory of Strongly-Coupled Quantum Matter Physics, Department of Physics, University of Science and Technology of China, 230026 Hefei, China

**Keywords:** Gels and hydrogels, Gels and hydrogels, Polymers

## Abstract

Living matter has the ability to perceive multiple stimuli and respond accordingly. However, the integration of multiple stimuli-responsiveness in artificial materials usually causes mutual interference, which makes artificial materials work improperly. Herein, we design composite gels with organic‒inorganic semi-interpenetrating network structures, which are orthogonally responsive to light and magnetic fields. The composite gels are prepared by the co-assembly of a photoswitchable organogelator (Azo-Ch) and superparamagnetic inorganic nanoparticles (Fe_3_O_4_@SiO_2_). Azo-Ch assembles into an organogel network, which shows photoinduced reversible sol-gel transitions. In gel or sol state, Fe_3_O_4_@SiO_2_ nanoparticles reversibly form photonic nanochains via magnetic control. Light and magnetic fields can orthogonally control the composite gel because Azo-Ch and Fe_3_O_4_@SiO_2_ form a unique semi-interpenetrating network, which allows them to work independently. The orthogonal photo- and magnetic-responsiveness enables the fabrication of smart windows, anti-counterfeiting labels, and reconfigurable materials using the composite gel. Our work presents a method to design orthogonally stimuli-responsive materials.

## Abstract (web summary)

The integration of multiple stimuli-responsiveness in artificial materials usually causes mutual interference, which makes artificial materials work improperly. Here, the authors design composite gels with organic‒inorganic semi-interpenetrating network structures, which are orthogonally responsive to light and magnetic fields.

## Introduction

Stimuli-responsive materials are smart materials that are responsive to external stimuli such as light^1–4^, electric^5^ and magnetic fields^6,7^, temperature^8,9^, humidity^10,11^, or pH^12–15^. Stimuli-responsive materials are widely studied because they exhibit potential applications in robotics^16^, biomedicine^17,18^, self-healing^19–21^, photonics^22^, etc. Recently, photonic materials and devices have been developed using stimuli-responsive materials^22^. For example, magnetic-responsive photonic crystals have been developed using superparamagnetic nanoparticles^23,24^, which have been used in display^25^ and bioassays^26^. In addition, photonic materials and devices can also be fabricated by photoresponsive materials that change optical properties under light irradiation. Such photoresponsive materials have been used for information storage^27,28^, anti-counterfeiting^29^, and smart windows^30^.

Although numerous stimuli-responsive materials have been developed, most of them are responsive to a single stimulus. In comparison, living matter can perceive multiple stimuli and respond accordingly. Inspired by living matter, the integration of multiple stimuli-responsiveness in artificial materials becomes a strategy for the design of smart materials. However, the integration of multiple stimuli-responsiveness in artificial materials usually causes mutual interference, which makes artificial materials function improperly. Therefore, it is a challenge to develop smart materials with multiple stimuli-responsiveness that can be controlled orthogonally.

**Fig. 1:**
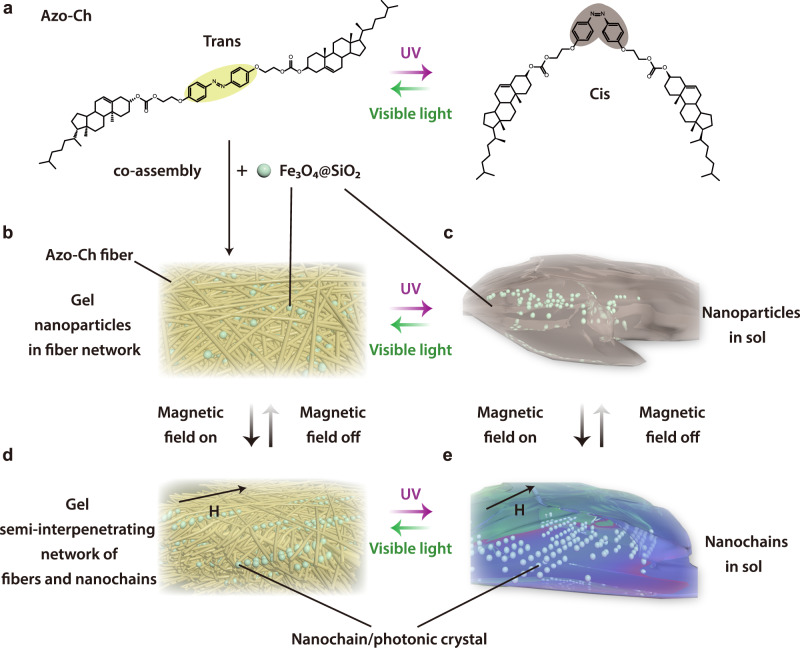
Schematic illustration of the orthogonally photo- and magnetic-responsive composite gel. **a** Chemical structure of the photoresponsive organic gelator (Azo-Ch) and cis-trans photoisomerization. **b** Composite gel prepared by the co-assembly of Azo-Ch and superparamagnetic Fe_3_O_4_@SiO_2_ nanoparticles in a solvent. The solvent cyclopentanone in the composite gel is omitted for clarity. **c** Fe_3_O_4_@SiO_2_ nanoparticles in the sol of cis Azo-Ch. **d** Semi-interpenetrating network of Azo-Ch fibers and Fe_3_O_4_@SiO_2_ nanochains. The nanochains exhibit photonic crystal structures, which show structural colors. H is the vector of the magnetic field. **e** Fe_3_O_4_@SiO_2_ nanochains in the sol of cis Azo-Ch. H is the vector of the magnetic field.

Herein, we report the design of a composite gel with an organic‒inorganic semi-interpenetrating network structure that is orthogonally responsive to light and magnetic fields. The design of the composite gel is inspired by semi-interpenetrating polymer networks^31^. The composite gel is prepared by the co-assembly of a photoreponsive organogelator (Azo-Ch) and superparamagnetic Fe_3_O_4_@SiO_2_ nanoparticles (Fig. 1). Azo-Ch assembles into supramolecular fibers that form a gel network; Fe_3_O_4_@SiO_2_ nanoparticles are located in the voids of the network (Fig. 1b). Azo-Ch shows reversible sol-gel transitions via cis-trans photoisomerization of the azobenzene group^32^. In both the gel and sol states, Fe_3_O_4_@SiO_2_ nanoparticles can reversibly form photonic nanochains via magnetic control (Fig. 1b–e). Azo-Ch and Fe_3_O_4_@SiO_2_ form a unique organic‒inorganic semi-interpenetrating network, which is different from conventional semi-interpenetrating networks that consist of crosslinked polymers with interpenetrated linear polymers^31^. The organic‒inorganic semi-interpenetrating network reported here consists of a network of organic fibers interpenetrated with inorganic nanochains, which expands the concept of semi-interpenetrating networks. The organic‒inorganic semi-interpenetrating network presents a unique network structure, which allows orthogonal control of its functions with light and magnetic fields. We demonstrate that smart photonic materials can be fabricated based on the orthogonal photo- and magnetic-responsiveness of the organic‒inorganic semi-interpenetrating network.

## Results

### Photoresponsive organogels of Azo-Ch

**Fig. 2:**
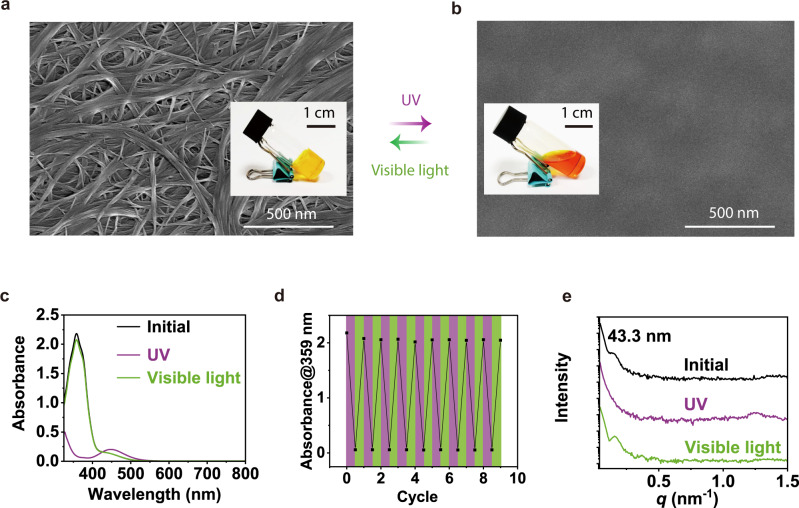
Photoisomerization of Azo-Ch and photoinduced reversible sol-gel transitions. **a, b** SEM images of the Azo-Ch gel and sol. Scale bars: 500 nm. Insets: photographs of the Azo-Ch gel and sol. Scale bars: 1 cm. **c** UV–vis absorption spectra of the Azo-Ch gel before irradiation (initial), after UV irradiation (365 nm, 12 mW cm^−2^, 40 s) and subsequent visible light irradiation (530 nm, 15 mW cm^−2^, 60 s). **d** Absorption changes of the Azo-Ch gel under alternating UV (365 nm, 12 mW cm^−2^, 40 s) and visible light (530 nm, 15 mW cm^−2^, 60 s) irradiation for 9 cycles. **e** SAXS profiles of the Azo-Ch gel before irradiation (initial), after UV irradiation (365 nm, 30 mW cm^−2^, 1 min) and subsequent visible light irradiation (530 nm, 40 mW cm^−2^, 5 min).

A component of the composite gel is the photoswitchable organic gelator Azo-Ch. Before we studied the composite gel of Azo-Ch and Fe_3_O_4_@SiO_2_ nanoparticles, we studied the organogel of Azo-Ch. Azo-Ch was synthesized via a multistep route and characterized using nuclear magnetic resonance (NMR) spectroscopy, ^13^C NMR spectroscopy, and mass spectrometry (Supplementary Figs. 1–10). The organogel was prepared by dissolving Azo-Ch in cyclopentanone at 70 °C and cooling it to room temperature (Fig. 2a). Gelation occurred because Azo-Ch self-assembled into fibers that formed a network (Supplementary Figs. 11 and 13). The average diameter of the fibers measured by scanning electron microscopy (SEM) and transmission electron microscopy (TEM) was 46 nm (Supplementary Figs. 12 and 14).

The azobenzene group endows Azo-Ch with photoresponsiveness, which was studied using UV‒vis absorption spectroscopy (Fig. 2c and Supplementary Fig. 15). The Azo-Ch gel exhibited a strong π–π* band at 359 nm and a weak n–π* band at 449 nm. Under UV irradiation, the π–π* band decreased, and the n–π* band increased, demonstrating that trans-to-cis isomerization occurred. Subsequent visible light irradiation transformed cis Azo-Ch back to the trans isomer. Azo-Ch was cycled 9 times by alternating UV and visible light irradiation, which showed that cis-trans photoisomerization was reversible (Fig. 2d).

In addition, photoisomerization of Azo-Ch resulted in reversible sol-gel transitions (Fig. 2a, b). Upon UV irradiation, the Azo-Ch gel changed to a sol, and the fiber network disappeared. Subsequent visible light irradiation induced the reformation of the gel. The photoinduced gel-to-sol transition was also studied using rheology (Supplementary Fig. 16). The storage modulus (G′) of the Azo-Ch gel was higher than the loss modulus (Gʺ). Upon UV irradiation, both G′ and Gʺ decreased immediately, suggesting that the gel became softer. Eventually, G′ was lower than Gʺ after UV irradiation, suggesting that a gel-to-sol transition occurred.

Moreover, small-angle X-ray scattering (SAXS) measurements were conducted to study the photoinduced sol-gel transition (Fig. 2e). The Azo-Ch gel had a peak at *q* = 0.14 nm^−1^, which corresponds to a period of 43.3 nm. This period is in accordance with the typical width of Azo-Ch fibers measured by SEM or TEM. Upon UV irradiation, the peak at *q* = 0.14 nm^−1^ disappeared. Upon subsequent visible light irradiation, the peak at *q* = 0.14 nm^−1^ appeared again. SAXS data revealed that the reversible sol-gel transitions were accompanied by the disappearance and reformation of Azo-Ch fibers. Furthermore, we noticed that an Azo-Ch xerogel, which was prepared by removing the solvent of an Azo-Ch organogel, showed fine structures in X-ray diffraction (XRD) (Supplementary Fig. 17). Three diffraction peaks corresponded to periods of 4.22, 2.09, and 1.41 nm, which are in the ratio of 1:1/2:1/3. Considering that the length of an Azo-Ch molecule was 4.34 nm, Azo-Ch formed a lamellar structure with an interlayer distance of 4.34 nm (Supplementary Fig. 18).

### Superparamagnetic Fe_3_O_4_@SiO_2_ nanoparticles

**Fig. 3:**
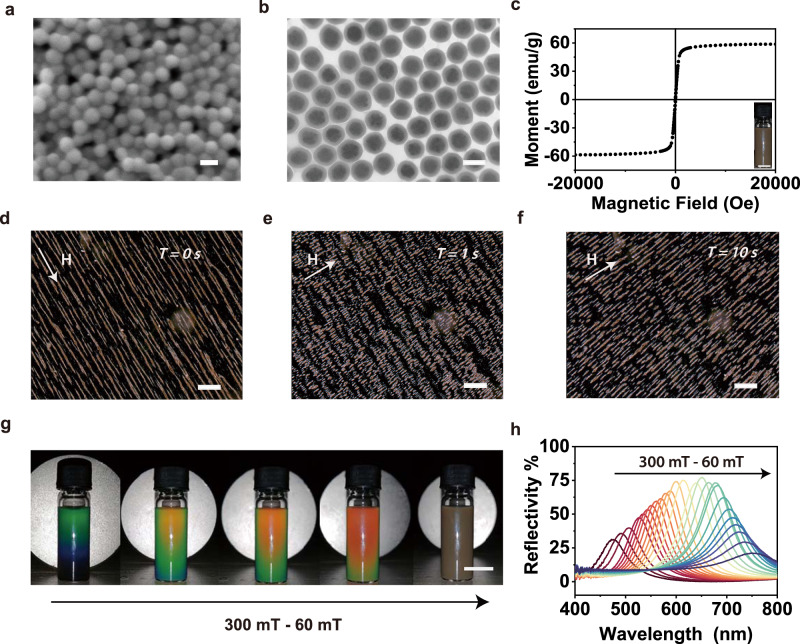
Characterization of superparamagnetic Fe3O4@SiO2 nanoparticles. **a, b** SEM and TEM images of Fe_3_O_4_@SiO_2_ nanoparticles. Scale bars: 200 nm. **c** Hysteresis curve of Fe_3_O_4_@SiO_2_ nanoparticles. Inset: Fe_3_O_4_@SiO_2_ nanoparticles dispersed in ethanol. Scale bar: 0.5 cm. **d–f** Dark-field optical microscopy images of Fe_3_O_4_@SiO_2_ nanoparticles during the change in the magnetic field at different time periods (0, 1, and 10 s). Scale bars: 200 ${{{{{\rm{\mu }}}}}}$m. **H** is the vector of the magnetic field. **g** Photographs of an ethanol dispersion of Fe_3_O_4_@SiO_2_ nanoparticles in response to a magnetic field. From left to right, the distance between the magnet and the dispersion gradually increased. Scale bar: 1 cm. **h** Reflection spectra of the dispersion of Fe_3_O_4_@SiO_2_ nanoparticles under magnetic fields with different intensities.

Another component of the composite gel is Fe_3_O_4_@SiO_2_ nanoparticles. Before we studied the composite gel of Azo-Ch and Fe_3_O_4_@SiO_2_ nanoparticles, we studied Fe_3_O_4_@SiO_2_ nanoparticles. Fe_3_O_4_@SiO_2_ nanoparticles were synthesized by a solvothermal reaction and subsequent hydrolysis of tetraethyl orthosilicate to form core-shell nanostructures (Supplementary Information). SEM images showed that Fe_3_O_4_@SiO_2_ nanoparticles had an average diameter of 165 nm, and TEM images revealed that the average diameter of the Fe_3_O_4_ core and the thickness of the SiO_2_ shell were 135 and 27 nm, respectively (Fig. 3a, b and Supplementary Fig. 19). The Fe_3_O_4_@SiO_2_ nanoparticles showed no remanence or coercivity in the hysteresis curve, which suggests that they are superparamagnetic (Fig. 3c). The mass magnetization saturation value of Fe_3_O_4_@SiO_2_ nanoparticles reached 58.6 emu g^−1^, which enabled their assembly using magnetic fields.

In a magnetic field, Fe_3_O_4_@SiO_2_ nanoparticles aligned into nanochains that were parallel to the magnetic field (Fig. 3d). When the magnetic field changed to a new direction, the nanoparticles moved along the new direction and reformed nanochains that reoriented along the new direction (Fig. 3e, f, Supplementary Fig. 20, and Movie 1).

The nanochains exhibited the periodic structures of one-dimensional photonic crystals, which showed structural colors (Fig. 3g, h and Supplementary Movie 2). The structural color changed from blue to red as the intensity of the magnetic field decreased. The tunable structural colors can be explained using Bragg’s equation:

1 $$\lambda=2{nd}{{\sin }}\theta$$

where *λ* is the wavelength of light, *n* refers to the effective refractive index, *d* represents the center-to-center distance of two adjacent Fe_3_O_4_@SiO_2_ nanoparticles, and *θ* is the angle between the incident light and diffraction plane. We interpret that *λ* was redshifted as the intensity of the magnetic field decreased because *d* increased.

### Organic‒inorganic composite gels with orthogonal photo- and magnetic-responsiveness

**Fig. 4:**
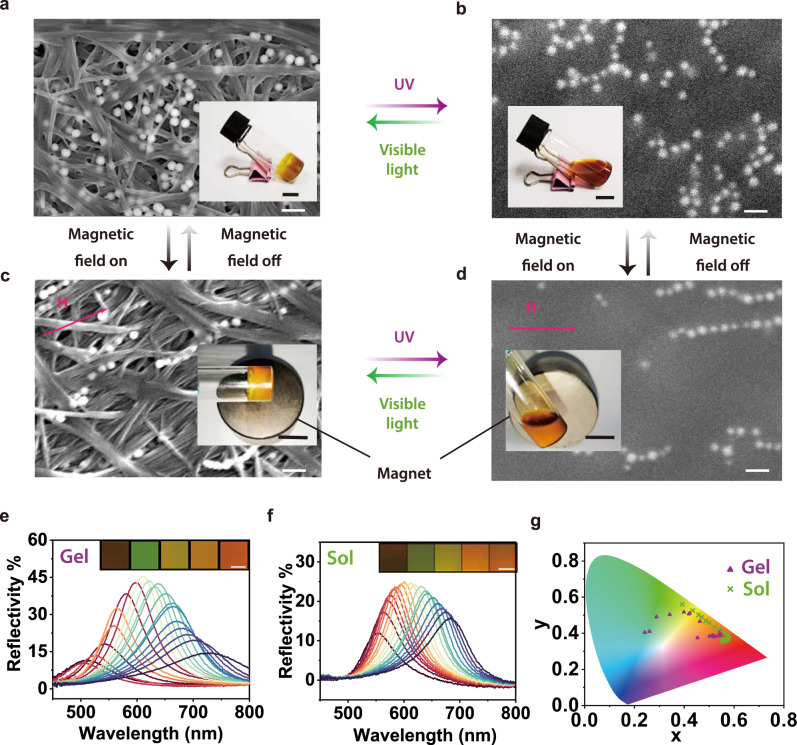
Orthogonally photo- and magnetic-responsive composite gel of Azo-Ch and Fe3O4@SiO2 nanoparticles. **a**–**d** SEM images and photographs of composite gel or sol under the control of light or magnetic field. Reversible sol-gel transitions were induced by light; reversible formation of nanochains was induced by a magnetic field. The scale bars for the SEM images and photographs are 500 nm and 1 cm, respectively. H: the vector of the magnetic field (100 mT). **e**–**g** Reflection spectra and CIE-1931 chromaticity diagram of composite gel and sol under magnetic fields with different intensities (300 to 60 mT). Insets in **e** and **f** show the colors of composite gel or sol. Scale bars: 0.5 cm.

To prepare an organic‒inorganic composite gel, Azo-Ch and Fe_3_O_4_@SiO_2_ nanoparticles were co-assembled in cyclopentanone. SEM images showed that Fe_3_O_4_@SiO_2_ nanoparticles were located in the voids of the network of Azo-Ch fibers (Fig. 4a). The nanocomposite gel showed photoinduced reversible sol-gel transitions when the magnetic field was either on or off; reversible nanoparticle-to-nanochain transitions were induced by switching the magnetic field on and off in both the gel and sol states (Fig. 4a–d). The nanochains of Fe_3_O_4_@SiO_2_ interpenetrated with the network of Azo-Ch fibers, which is an analog of conventional semi-interpenetrating networks that are composed of crosslinked polymers with interpenetrated linear polymers. The size of our semi-interpenetrating network is larger than that of a conventional semi-interpenetrating network of polymers. In our semi-interpenetrating network, both Azo-Ch and Fe_3_O_4_@SiO_2_ have sufficient space for independent structural rearrangements. Therefore, the photo- and magnetic-responsiveness of the semi-interpenetrating network is orthogonal.

Light can induce photochromism of Azo-Ch, and a magnetic field can induce the formation of photonic nanochains of Fe_3_O_4_@SiO_2_. Thus, the color of the composite gel can be controlled by light or a magnetic field (Fig. 4e, f). The color of the composite gel changed from brown to green, yellow, orange and red as the intensity of the magnetic field decreased. The composite gel was switched to composite sol via UV irradiation (Supplementary Fig. 21). The colors of the composite gel and sol are in different ranges of the chromaticity diagram (Fig. 4g). Such a difference is in line with the theoretical analyses, which suggests that Azo-Ch provides a suitable matrix for the formation of photonic nanochains (Supplementary Figs. 22–26, Supplementary Table 1, and Supplementary Movies 3 and 4).

We compare our composite gel with covalent gels that contain superparamagnetic nanochains. Covalent gels have been used as matrices to fix nanochains^25,26^. In contrast, Azo-Ch forms a responsive network, which provides a dynamic environment for the rearrangement and reversible control of the superparamagnetic nanoparticles. Therefore, the structural colors in the composite gel are not fixed but tunable, which makes the composite gel different from covalent gels with photonic nanochains.

Moreover, the dynamic features and orthogonally responsive functions of our semi-interpenetrating network enable the fabrication of smart photonic materials, which will be presented below.

### Smart windows based on composite gel

**Fig. 5:**
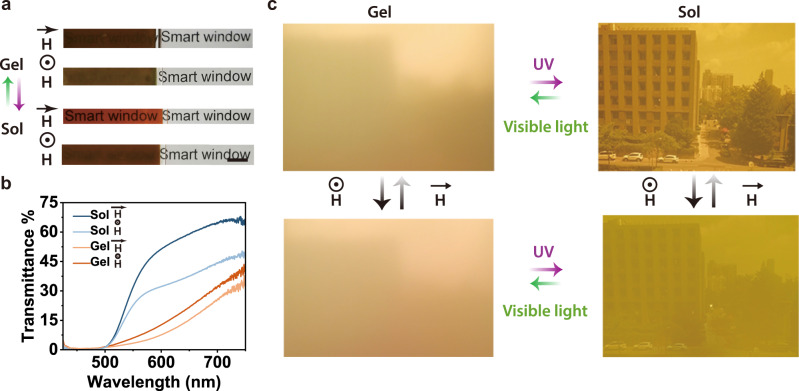
Photo- and magnetic-responsive smart windows. **a** Photographs of a smart window controlled by light and a magnetic field. Scale bar: 0.5 cm. **b** Transmission spectra of a smart window controlled by light and a magnetic field. **c** Photographs captured through a smart window at four different states.

To prepare smart windows, composite gels were sandwiched between two glasses (Fig. 5 and Supplementary Figs. 27 and 28). Sealed glasses prevented the evaporation of solvents (Supplementary Fig. 29). The transparency of the smart window was controlled by light and magnetic fields (Fig. 5a). Light irradiation induced photochromism of Azo-Ch and modulated the scattering due to the sol-gel transition. The magnetic field modulated the reflection and scattering of Fe_3_O_4_@SiO_2_ nanochains. Thus, the smart window was switchable among four states under the control of light and magnetic field (Fig. 5b, c and Supplementary Movies 5 and 6).

According to some recently reported smart windows fabricated using other materials^33–35^, we showed the characteristics of our smart window here. The switching times of the smart window by magnetic fields and light are ~2 s and 2 min, respectively, which are fast. The transmittance of the smart window in the visible range is up to 60%, and some undesirable short-wavelength light can be filtered.

### Rewritable multicolor patterns

**Fig. 6:**
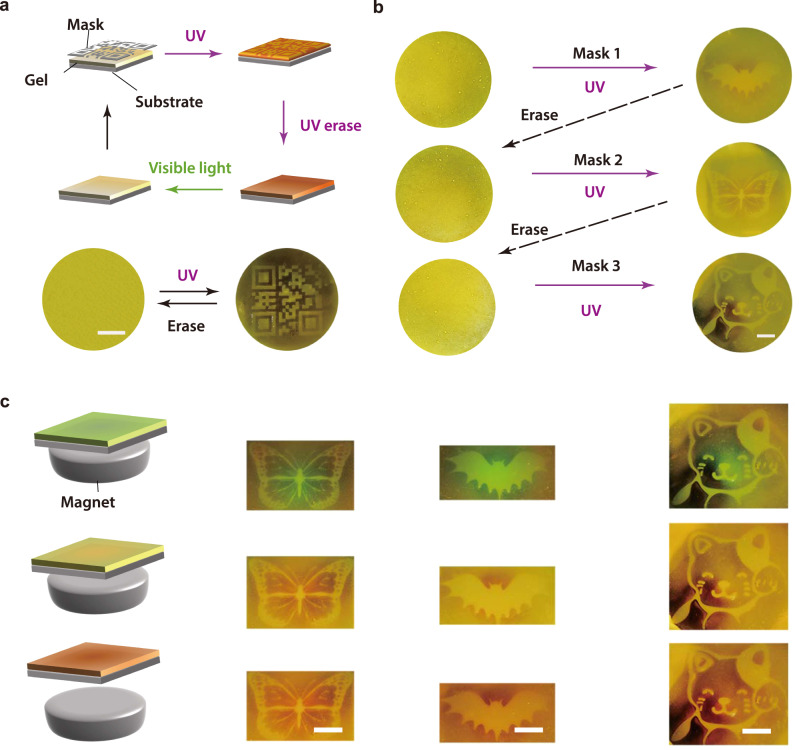
Rewritable multicolor patterns. **a** Schematic illustration and photographs of writing and erasing a QR code on a composite gel. UV (365 nm, 30 mW cm^−2^, 30 s) and visible light (530 nm, 40 mW cm^−2^, 5 min) were used for writing and erasing procedures. Scale bar: 1 cm. **b** Photographs showing (re)writing and erasing different patterns. Scale bar: 1 cm. **c** Schemes and photographs of patterns in response to magnetic fields (50–300 mT). Scale bars: 1 cm.

We prepared rewritable multicolor patterns using composite gels, which can be used for anti-counterfeiting and storing temporary or sensitive information. To write a pattern, a composite gel was irradiated with UV light through a photomask (Fig. 6a). Photoisomerization and gel-to-sol transition occurred at the irradiated areas, which produced a pattern. To erase the pattern, the whole sample was irradiated with UV light and subsequent visible light. We wrote and erased a QR code via the abovementioned procedure.

We rewrote and erased bat-, butterfly-, and cat-shaped patterns on a composite gel (Fig. 6b). More complex patterns can be also fabricated on the composite gel (Supplementary Fig. 30). Additionally, the colors of the patterns can be modulated using a magnetic field because Fe_3_O_4_@SiO_2_ nanoparticles formed photonic crystals (Fig. 6c and Supplementary Fig. 31). Moreover, the patterns on a composite gel can be re-written for multi cycles (Supplementary Fig. 32). The synergism of photopatterning and magnetic-responsive structural colors can improve the level of security for storing sensitive or temporary information.

### Reconfigurable photonic materials

**Fig. 7:**
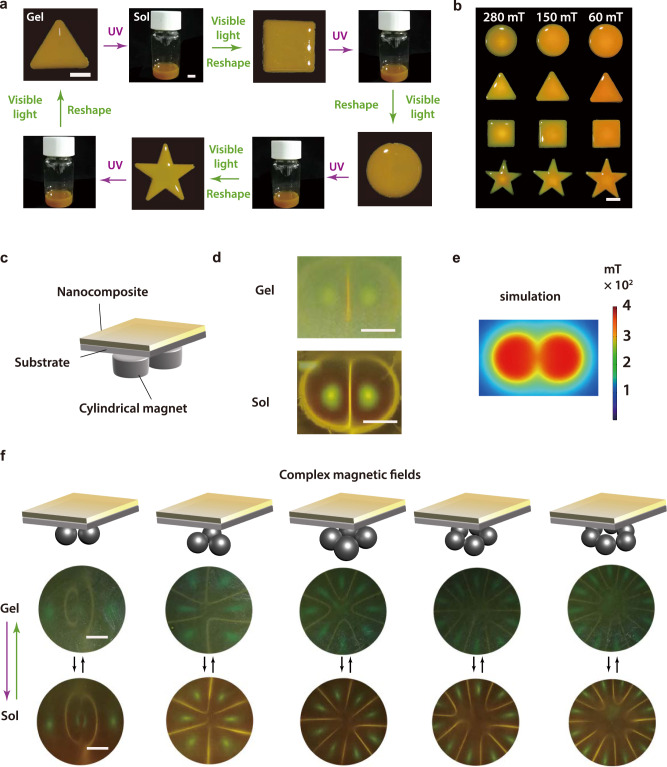
Reconfiguring composite gels with light and magnetic fields. **a** Reshaping composite gel with light. UV (365 nm, 30 mW cm^−2^, 5 min) and visible light (530 nm, 40 mW cm^−2^, 20 min) were used for reshaping. Scale bars: 1 cm. **b** Photographs of composite gels with different shapes under magnetic fields with different intensities. Scale bar: 1 cm. **c**, **d** Scheme and photographs of a composite gel or sol in response to two cylindrical magnets. **e** Computer simulation of the distribution of magnetic flux created by two cylindrical magnets. **f** Schematic illustration and photographs of composite gel or sol under complex magnetic fields. Scale bar: 0.5 cm.

A unique feature of the composite gel is that it is reconfigurable under the stimulation of light and magnetic fields. A triangle-shaped gel was prepared by gelation in a triangle mold (Fig. 7a). It was reshaped to a square, circle, and pentagram via photoinduced reversible sol-gel transitions using different molds. The composite gels with different shapes exhibited structural colors under magnetic fields (Fig. 7b).

Additionally, photonic patterns with spatially distributed structural colors are reconfigurable by magnetic fields. When two cylindrical magnets were placed underneath a composite gel or sol for 0.1 s, photonic patterns appeared (Fig. 7c, d). Computer simulation revealed that the photonic patterns were attributed to the spatial distribution of the magnetic flux (Fig. 7e). Furthermore, we used different magnet assemblies to induce different photonic patterns (Fig. 7f). These results showed that the composite gels can be used as reconfigurable photonic crystals.

## Discussion

In this study, we developed a composite gel by the co-assembly of a photoresponsive gelator Azo-Ch and superparamagnetic Fe_3_O_4_@SiO_2_ nanoparticles. The composite gel exhibits a unique organic‒inorganic semi-interpenetrating network structure, which allows orthogonal control of Azo-Ch and Fe_3_O_4_@SiO_2_ nanoparticles with light and magnetic fields. We designed smart windows, rewritable multicolor patterns, and reconfigurable photonic materials based on the orthogonal stimuli-responsiveness of the composite gel.

We compare our semi-interpenetrating network with conventional semi-interpenetrating networks of polymers. Conventional semi-interpenetrating networks of polymers consist of a crosslinked polymer network with interpenetrated linear polymer chains^31^. In contrast, our semi-interpenetrating network consists of a network of organic gelators and inorganic nanoparticles.

Our study expands the range of semi-interpenetrating networks, which provides interesting network structures and organic‒inorganic materials for fundamental research. We envision that polymers in conventional (semi-)interpenetrating networks can be replaced by a variety of inorganic, organic, and composite materials, which can endow (semi-)interpenetrating networks with designed properties and functions. Our work also shows that the construction of semi-interpenetrating networks is a method for the design of smart materials with orthogonal stimuli-responsiveness.

## Methods

The details for the synthesis, preparation and characterization of Azo-Ch, Fe_3_O_4_@SiO_2_ nanoparticles, and the composite are provided in the Supplementary Information. The reflection spectra were measured using an Ocean Optics USB-4000 spectrometer. The colors in the CIE-1931 chromaticity diagram were obtained using the reflection spectra. Each point in the CIE-1931 chromaticity diagram corresponds to a spectrum. The details of the DFT calculations (atom-centered basis function) and COMSOL Multiphysics simulations are also provided in the Supplementary Information. The diameters of the fibers of Azo-Ch and Fe_3_O_4_@SiO_2_ nanoparticles were obtained by statistically analyzing TEM and SEM images. The lengths of the Fe_3_O_4_@SiO_2_ nanochains in the composite gel and sol at different time periods were obtained by statistically analyzing the optical microscopy images (Supplementary Information).

## Supplementary information

Supplementary Information

Description of Additional Supplementary Files

Supplementary Movie 1

Supplementary Movie 2

Supplementary Movie 3

Supplementary Movie 4

Supplementary Movie 5

Supplementary Movie 6

## Data Availability

The authors declare that the data supporting the findings of this study are available within the paper and its supplementary information files or the data are available from the corresponding authors upon request.
