# Supplementary material for: Organic‒inorganic semi-interpenetrating networks with orthogonal light- and magnetic-responsiveness for smart photonic gels
Source: Nat Commun. 2023 Feb 22;14:1000. doi: 10.1038/s41467-023-36706-7 (PMC9946997; doi:10.1038/s41467-023-36706-7)
Supplement: Supplementary file 1 — Supplementary Information [file 41467_2023_36706_MOESM1_ESM.pdf]

Supplementary Information

Organic–inorganic semi-interpenetrating networks with orthogonal light- and magnetic-responsiveness for smart photonic gels

Minghao Wang[1], Chen Nie[1], Junbang Liu[2], Si Wu[1]*

[1]CAS Key Laboratory of Soft Matter Chemistry, Anhui Key Laboratory of Optoelectronic Science and Technology, Department of Polymer Science and Engineering, University of Science and Technology of China, Hefei 230026, China

[2]CAS Key Laboratory of Strongly-Coupled Quantum Matter Physics, Department of Physics, University of Science and Technology of China, Hefei 230026, China

*E-mail: siwu@ustc.edu.cn

Table of contents

Materials 5

Instruments and Characterization 5

DFT calculations 6

COMSOL Multiphysics simulation 7

Synthesis 7

Supplementary Figure 1. Synthetic route for Azo-Ch 7

Synthesis of 4,4'-dihydroxyazobenzene 8

Synthesis of 4,4′-di(2-hydroxyethoxy)azobenzene 8

Synthesis of Azo-Ch 9

Synthesis of Fe_3O_4 nanoparticles 10

Synthesis of Fe_3O_4@SiO_2 nanoparticles 10

Preparation of an Azo-Ch gel 10

Preparation of a composite gel 11

Fabrication of a smart window 11

Supplementary Figure 2. ^{1}H NMR spectrum of 4,4'-dihydroxyazobenzene 12

Supplementary Figure 3. ^{13}C NMR spectrum of 4,4'-dihydroxyazobenzene 12

Supplementary Figure 4. MS spectrum of 4,4'-dihydroxyazobenzene 13

Supplementary Figure 5. ^{1}H NMR spectrum of 4,4′-di(2-hydroxyethoxy) azobenzene 13

Supplementary Figure 6. ^{13}C NMR spectrum of 4,4′-di(2-hydroxyethoxy) azobenzene 14

Supplementary Figure 7. MS spectrum of 4,4′-di(2-hydroxyethoxy) azobenzene ... 15

Supplementary Figure 8. ^{1}H NMR spectrum of Azo-Ch 15

Supplementary Figure 9. ^{13}C NMR spectrum of Azo-Ch 16

Supplementary Figure 10. MS spectrum of Azo-Ch 16

Supplementary Figure 11. SEM images of the Azo-Ch gel 17

Supplementary Figure 12. The diameter distribution histogram of Azo-Ch fibers measured by SEM 17

Supplementary Figure 13. TEM images of the Azo-Ch gel....................................18

Supplementary Figure 14. The diameter distribution histogram of Azo-Ch fibers measured by TEM..18

Supplementary Figure 15. UV–vis absorption spectra of the Azo-Ch gel................19

Supplementary Figure 16. The rheology data of the Azo-Ch gel upon UV irradiation 19

Supplementary Figure 17. XRD pattern of the xerogel of Azo-Ch...........................20

Supplementary Figure 18. 3D structure of Azo-Ch calculated by density functional theory (DFT) 20

Supplementary Figure 19. TEM image and diameter distribution histograms of Fe_3O_4@SiO_2 nanoparticles 21

Supplementary Figure 20. Optical microscopy images of Fe_3O_4@SiO_2 nanoparticles under magnetic fields of different directions 22

Supplementary Figure 21. Rheology data of the composite gel upon UV irradiation 22

Supplementary Figure 22. Optical microscopy images showing the magnetic-induced formation of nanochains in composite sol 23

Supplementary Figure 23. Boxplots of Fe_3O_4@SiO_2 nanochains at different time periods...24

Supplementary Figure 24. Dark-field optical microscopy images showing magetic-induced formation of nanochains in composite gel 24

Supplementary Figure 25. Boxplots of Fe_3O_4@SiO_2 chains at different time periods 25

Supplementary Figure 26. Mean chain length $S(t)$ of Fe_3O_4@SiO_2 as a function of time t 25

Supplementary Figure 27. Photographs and transmission spectra of a 2 mm smart window...27

Supplementary Figure 28. Photographs and transmission spectra of a 10 mm smart window...27

Supplementary Figure 29. Weight change of a smart window for 20 days 28

Supplementary Figure 30. A photograph of a complex pattern on a composite gel 28
Supplementary Figure 31. Photographs of composite gels with different patterns under magnetic fields 29
Supplementary Figure 32. Photographs of rewriting a pattern on a composite gel for 20 times using UV and visible light...29
Supplementary Table 1. The length of Fe_3O_4@SiO_2 nanochains in composite gel and sol at different time periods 30
References...31

Materials

p-Aminophenol (>98%), sodium acetate (NaAc) (99%) and tetraethyl orthosilicate (TEOS) (98%) were purchased from Macklin. Phenol (AR), sodium hydroxide (NaOH) (AR), ammonium hydroxide (AR) and ethylene glycol (EG) (AR) were purchased from Sinopharm Chemical Reagent. Cholesteryl chloroformate (98%), 2-bromoethanol (98%), pyridine (99.5%, extra dry, water ≤ 50 ppm) and dichloromethane (99.9%, extra dry, water ≤ 50 ppm) were purchased from Energy Chemical. Iron chloride ($FeCl_3$) (99.9%) and poly(4-styrenesulfonic acid-co-maleic acid) sodium salt (PSSMA) (SS:MA = 1:1) were purchased from Aladdin. EG was predried over activated 4 Å molecular sieves before use.

Instruments and Characterization

^1H nuclear magnetic resonance (^1H NMR) and ^{13}C NMR spectra were recorded on a Bruker AVANCE III NMR spectrometer. Mass spectrometry (MS) data were obtained using Waters Q-Tof Premier spectrometer. UV–vis absorption spectra were measured using a Shimadzu UV-2600 spectrometer. Optical microscopy images were captured on an OLYMPUS BX51 microscope equipped with a CCD camera. Trans-to-cis photoisomerization was induced by an LED at λ = 365 nm (LCS-0365-07-22, Mightex Systems), and cis-to-trans photoisomerization was induced by an LED at λ = 530 nm (LCS-0530-15-22, Mightex Systems). The output power of the LEDs was controlled

by an LED controller (SLC-MA04-MU, Mightex Systems). X-ray diffraction (XRD) patterns were obtained on a Philips X'Pert PRO diffractometer using Cu Kα (λ= 1.5418 Å). Transmission electron microscopy (TEM) images were recorded using a Hitachi H-7650 system. The samples for TEM measurements were prepared by applying a drop of sample solution on a copper grid and leaving it dry in air. Scanning electron microscopy (SEM) images were collected using a Quanta FEG 250 system. The samples for SEM measurements were prepared by casting the samples on silicon wafers and leaving them dry in air. The reflection spectra were measured by an Ocean Optics USB-4000 spectrometer. Rheological measurements were conducted on a Waters TA 20 system. SAXS experiments were performed on an Anton Paar SAXSpoint 2.0 system using the small/wide angle X-ray scattering beamline. Samples were inserted into 1.0 mm borosilicate glass capillaries, which were sealed.

DFT calculations

The molecular length of Azo-Ch was calculated using the FHI-aims program (aims.210716). Frequency calculations were performed using density functional theory (DFT) (Method = PBE). The first tier basis set was used and high-angular momentum radial functions in second tier were added for auxiliary[1]. Basis set was numeric atom-centered basis function. The calculated molecular length of Azo-Ch was 43.4 Å.

COMSOL Multiphysics simulation

Magnetostatic calculations were performed using the AC/DC module of COMSOL Multiphysics modeling software (COMSOL Multiphysics 5.5) to investigate the local magnetic field distribution around two cylindrical magnets (diameter: 10 mm; height: 20 mm). The relative magnetic permeability of the magnet (4000) and the air (1) were incorporated into the model. The magnet is magnetized in the z direction (7.5×10^5 $A\cdot m^{-1}$). The geometry of the system was confined to a three-dimensional area (100 mm × 100 mm × 100 mm) with extremely fine magnets at the center of the chamber. The simulations were performed by solving Maxwell's equations with a fully coupled linear solver.

Synthesis

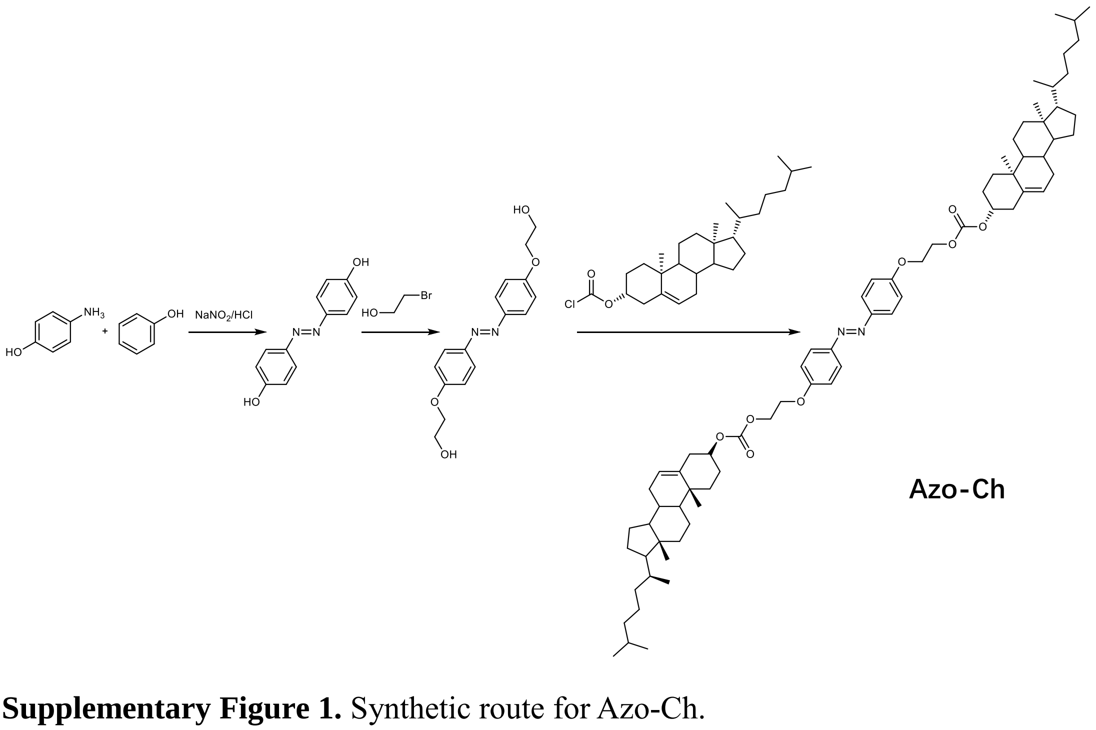

Supplementary Figure 1. Synthetic route for Azo-Ch.

Synthesis of 4,4'-dihydroxyazobenzene: p-Aminophenol (10.9 g, 0.1 mol) was dissolved in a mixture of hydrochloric acid (20 mL) and ice (32 g). To the above mixture, a solution containing water (28 mL), ice (28 g), and sodium nitrite (6.9 g, 0.1 mol) was added slowly. The mixture was stirred for 30 min at -5 to 0 °C. Then, an aqueous solution of phenol (9.4 g, 0.1 mol) in 2 mol·L^{-1} sodium hydroxide was added to the mixture. The reaction mixture was stirred for 8 h at pH 9-10. After neutralization with HCl, the resulting mixture was filtered and washed with water. The crude product was dried and purified by recrystallization from an ethanol/water mixture to obtain the compound as a violet solid. Yield: 60%. ^{1}H NMR (400 MHz, DMSO-d6), δ (ppm): 10.14 (s, 2H), 7.71 (d, J = 8.8 Hz, 4H), 6.90 (d, J = 8.8 Hz, 4H); ^{13}C NMR (100 MHz, DMSO-d6): δ 160.06 (1), 145.31 (4), 124.24 (3), 115.85 (2). ESI-MS (m/z), calcd. for $C_{12}H_{11}N_2O_2^+$ $[M-H]^+$, 215.1, found, 215.1.

Synthesis of 4,4′-di(2-hydroxyethoxy)azobenzene: K_2CO_3 (3.1 g, 0.022 mol) was added to a solution of 4,4'-dihydroxyazobenzene (2.1 g, 9.81 mmol) in DMF (30 mL). The solution was stirred for 30 min at 30 °C. Then, KI (0.08 g, 0.48 mmol) and 2-bromoethanol (2.8 g, 0.02 mol) were added to the solution. The reaction mixture was then stirred vigorously for 48 h at 160 °C. The resulting mixture was cooled to room temperature and poured into 400 g of crushed ice. The crude product was precipitated. The precipitate was filtered off and recrystallized twice from ethanol to give a yellow powder. Yield: 60%. ^{1}H NMR (400 MHz, DMSO-d6), δ (ppm): 7.83 (d, J = 8.9 Hz, 4H),

7.11 (d, J = 9.0 Hz, 4H), 4.94 (t, J = 5.1 Hz, 2H), 4.09 (t, J = 5.0 Hz, 4H), 3.75 (q, J = 5.0 Hz, 4H). ^{13}C NMR (100 MHz, DMSO-d6): δ 161.41 (1), 146.57 (2), 124.59 (3), 115.46 (4), 70.43 (5), 59.94 (6). ESI-MS (m/z) calcd. for $C_{16}H_{19}N_2O_4^+$ $[M-H]^+$, 303.1, found, 303.1.

Synthesis of Azo-Ch: 4,4′-Di(2-hydroxyethoxy)azobenzene (0.76 g, 2.51 mmol) was dissolved in anhydrous dichloromethane (20 mL) and pyridine (10 mL). Then, cholesteryl chloroformate (2.5 g, 5.56 mmol) dissolved in anhydrous dichloromethane (30 mL) was added dropwise, and the mixture was reacted for 24 h at room temperature. After that, the mixture was evaporated to dryness, and the obtained crude product was dissolved in chloroform and then precipitated in methanol. The product was purified by column chromatography (chloroform). Yield: 61%. ^{1}H NMR (400 MHz, $CDCl_3$), δ (ppm): 7.94 (d, J = 8.8 Hz, 4H), 7.01 (d, J = 8.9 Hz, 4H), 5.40 (d, J = 5.0 Hz, 1H), 4.51 (h, J = 4.4 Hz, 6H), 4.28 (t, J = 4.4 Hz, 4H), 2.46 – 2.38 (m, 4H), 2.03–0.85 (m, 76H), 0.67 (s, 6H). ^{13}C NMR (100 MHz, $CDCl_3$): δ 160.48 (1), 154.52 (2), 147.38 (3), 139.36 (4), 124.51 (5), 123.16 (6), 114.86 (7), 78.38 (8), 66.04 (9), 65.74 (10), 56.78 (11), 56.22 (12), 50.08 (13), 42.41 (14), 39.81 (15), 39.63 (16), 38.11 (17), 36.94 (18), 36.64 (19), 36.29 (20), 35.91 (21), 32.01 (22), 31.93 (23), 28.35 (24), 28.14 (25), 27.78 (26), 24.40 (27), 23.95 (28), 22.97 (29), 22.70 (30), 21.15 (31), 19.39 (32), 18.83 (33), 11.98 (34). ESI-MS (m/z) calcd. for $C_{72}H_{107}N_2O_8^+$ $[M-H]^+$,1127.8, found 1127.7.

Synthesis of Fe_3O_4 nanoparticles: $FeCl_3$ (0.38 g, 2.3 mmol), NaAc (1.5 g, 0.18 mmol), PSSMA (0.75 g, 3.3 mmol), and 50 μL deionized water were added to predried EG (20 mL). The solution was vigorously stirred for 15 min until there was no insoluble matter in the homogeneous yellow solution. Then, NaOH (0.3 g, 7.5 mmol) was added to the solution and stirred until there was no insoluble matter, and the color changed from light brown to black. Finally, the solution was transferred into a 25 mL Teflon-lined stainless-steel autoclave. Then, the autoclave was sealed and kept at 190 °C in an oven for 9 h. After it was naturally cooled to room temperature, the yielded Fe_3O_4 nanoparticles were collected by a magnet and washed with a mixture of ethanol and water three times. Fe_3O_4 nanoparticles were dispersed in water (15 mL).

Synthesis of Fe_3O_4@SiO_2 nanoparticles: Typically, a 12 mL water dispersion of Fe_3O_4 nanoparticles, 80 mL ethanol, and 4 mL ammonium hydroxide were mixed in a three-neck flask under a stirring rate of 500 rpm at 50 °C. After vigorous stirring for 10 min, 400 μL of TEOS was added to the above mixture at a rate of 100 μL every 20 min. After continuous stirring for 1 h, the nanoparticles were isolated by a magnet, rinsed with ethanol and water three times, and finally dispersed in ethanol (30 mL).

Preparation of an Azo-Ch gel

Azo-Ch (50 mg) was dissolved in 5 mL cyclopentanone at 70 °C and cooled to room temperature. The sample was placed in the dark overnight for gelation.

Preparation of a composite gel

A dispersion of Fe_3O_4@SiO_2 nanoparticles (10 mL, 8 mg·mL^{-1}) was centrifuged and added to an Azo-Ch solution (10 mL, 1 w/v % in cyclopentanone). The dispersion was sonicated for 20 min to yield a homogeneous dispersion. The dispersion was injected into a cuvette (path length: 1 mm), which was placed in the dark overnight for gelation.

Fabrication of a smart window

A dispersion of Fe_3O_4@SiO_2 nanoparticles (1 mL, 8 mg·mL^{-1}) was centrifuged and added to an Azo-Ch solution (2 mL, 1 w/v % in a mixture of cyclopentanone/methanol (7/3, v/v)). The dispersion was sonicated for 20 min to yield a homogeneous dispersion. The dispersion was injected into a cuvette (path length: 2 mm), which was placed in the dark overnight for the formation of a composite gel.

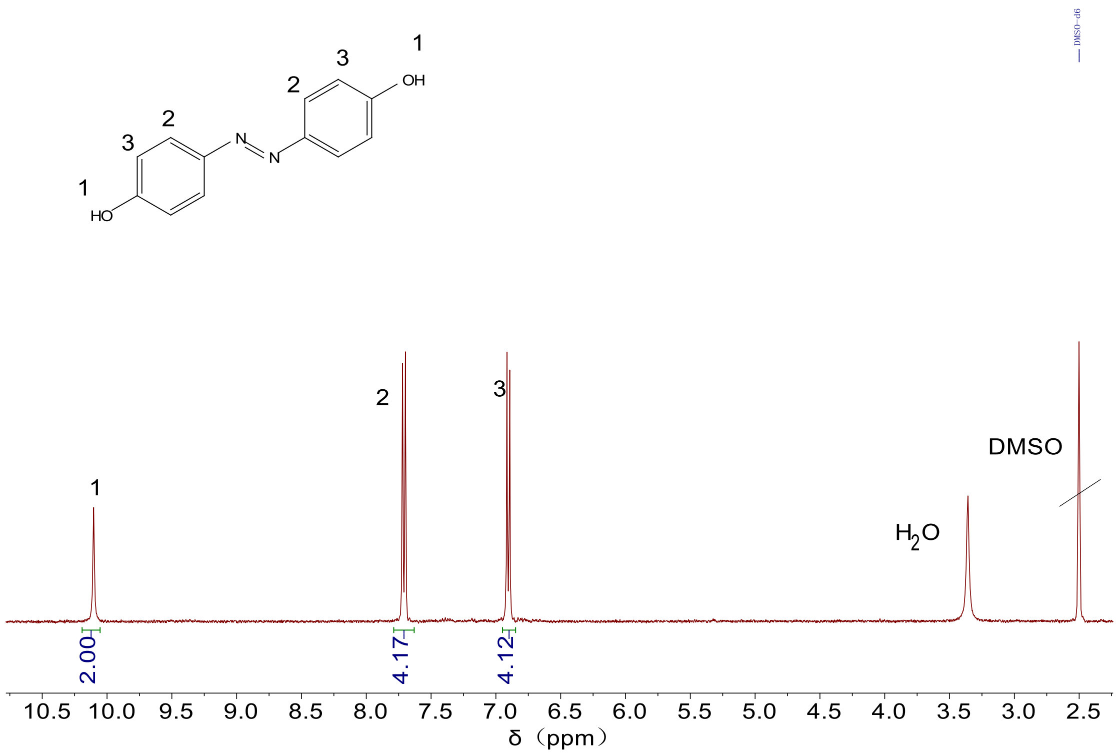

Supplementary Figure 2. ^{1}H NMR spectrum of 4,4'-dihydroxyazobenzene (400 MHz, DMSO-d6, 25 °C).

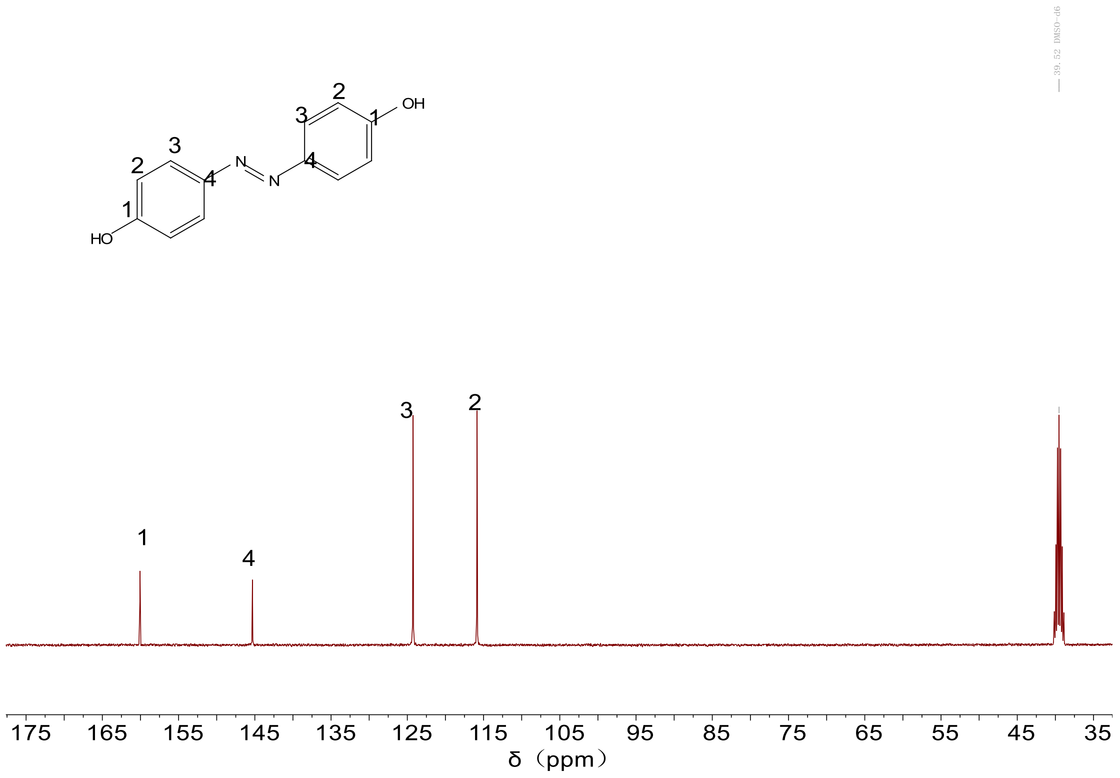

Supplementary Figure 3. ^{13}C NMR spectrum of 4,4'-dihydroxyazobenzene (100 MHz,

DMSO-d6, 25 °C).

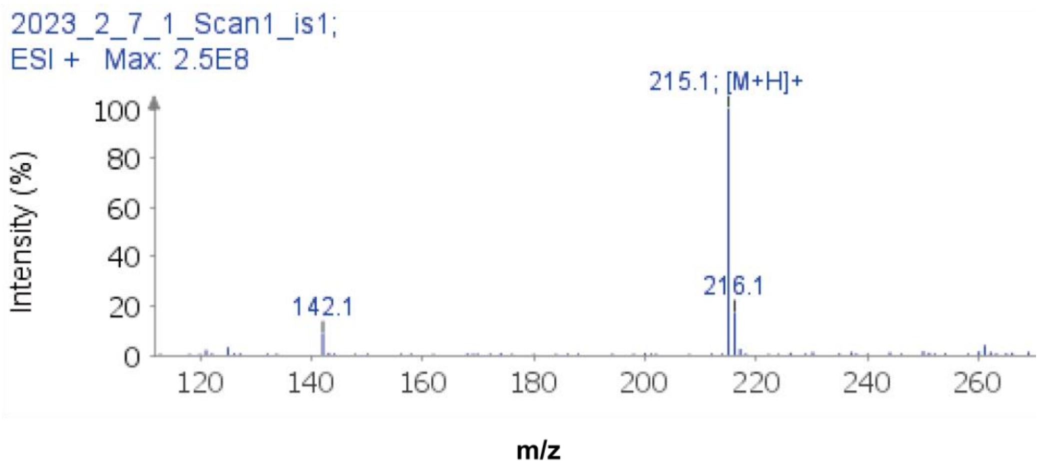

Supplementary Figure 4. MS spectrum of 4,4'-dihydroxyazobenzene.

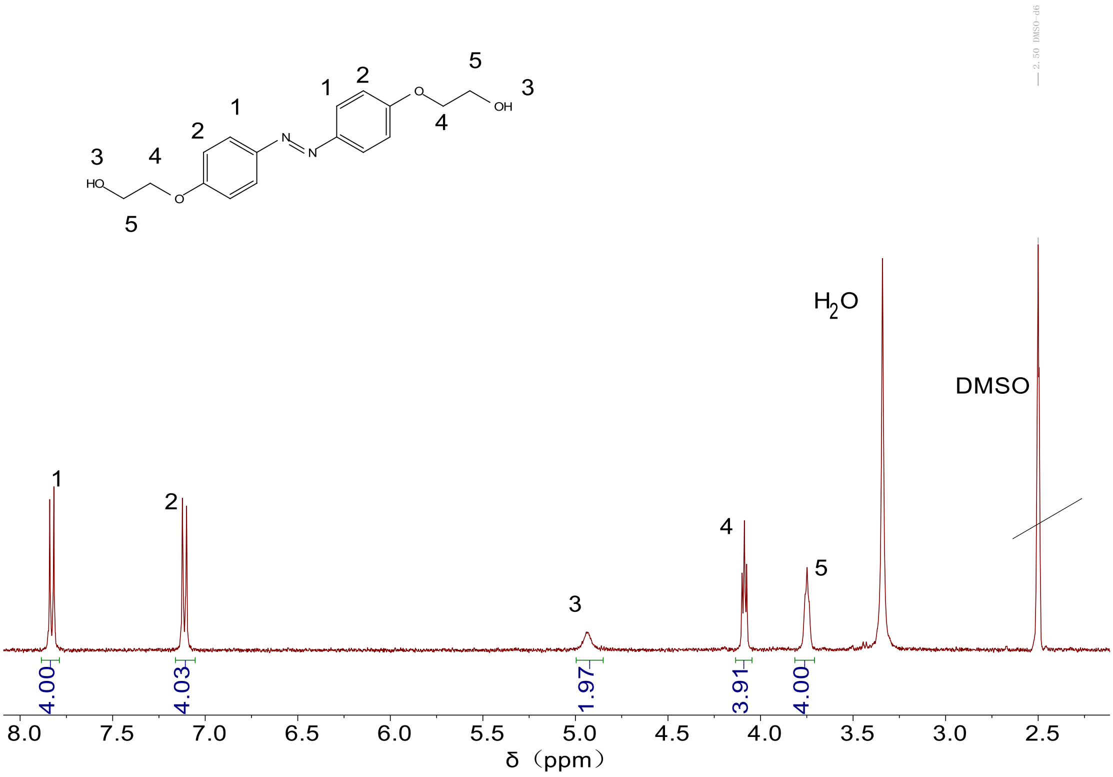

Supplementary Figure 5. ^{1}H NMR spectrum of 4,4′-di(2-hydroxyethoxy) azobenzene (400 MHz, DMSO-d6, 25 °C).

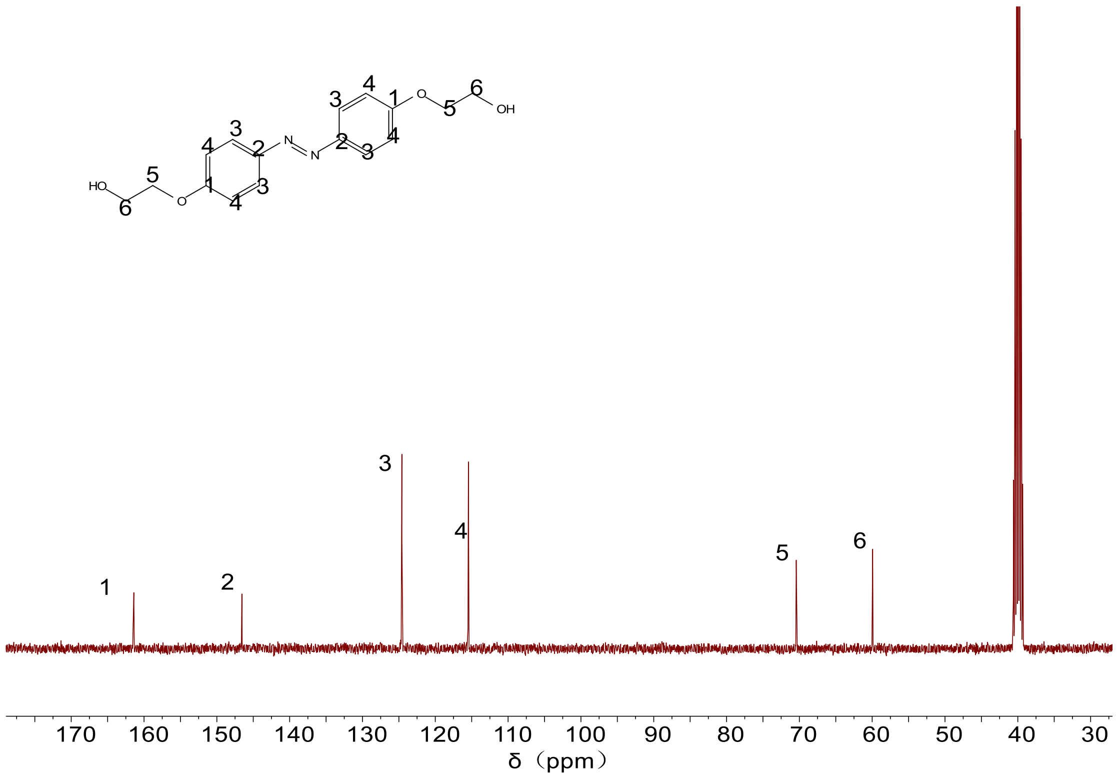

Supplementary Figure 6. ^{13}C NMR spectrum of 4,4′-di(2-hydroxyethoxy) azobenzene (100 MHz, DMSO-d6, 25 °C).

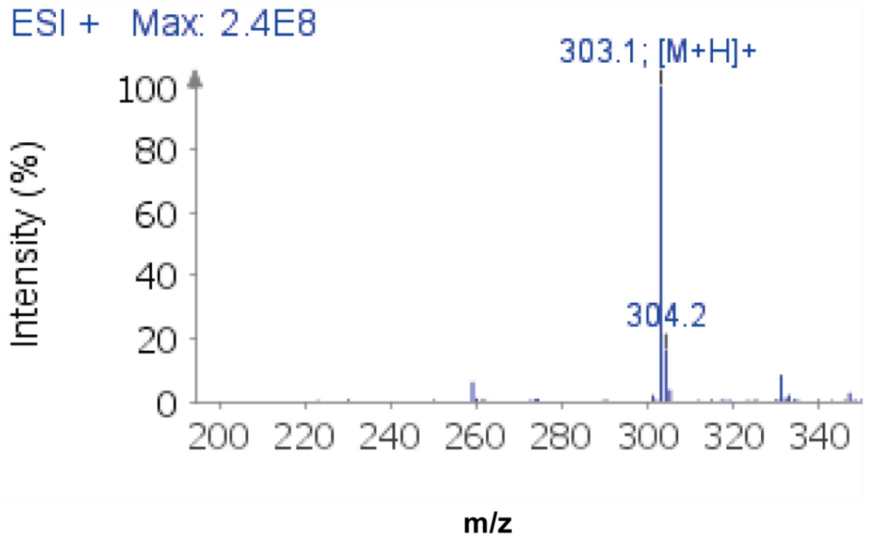

Supplementary Figure 7. MS spectrum of 4,4′-di(2-hydroxyethoxy) azobenzene.

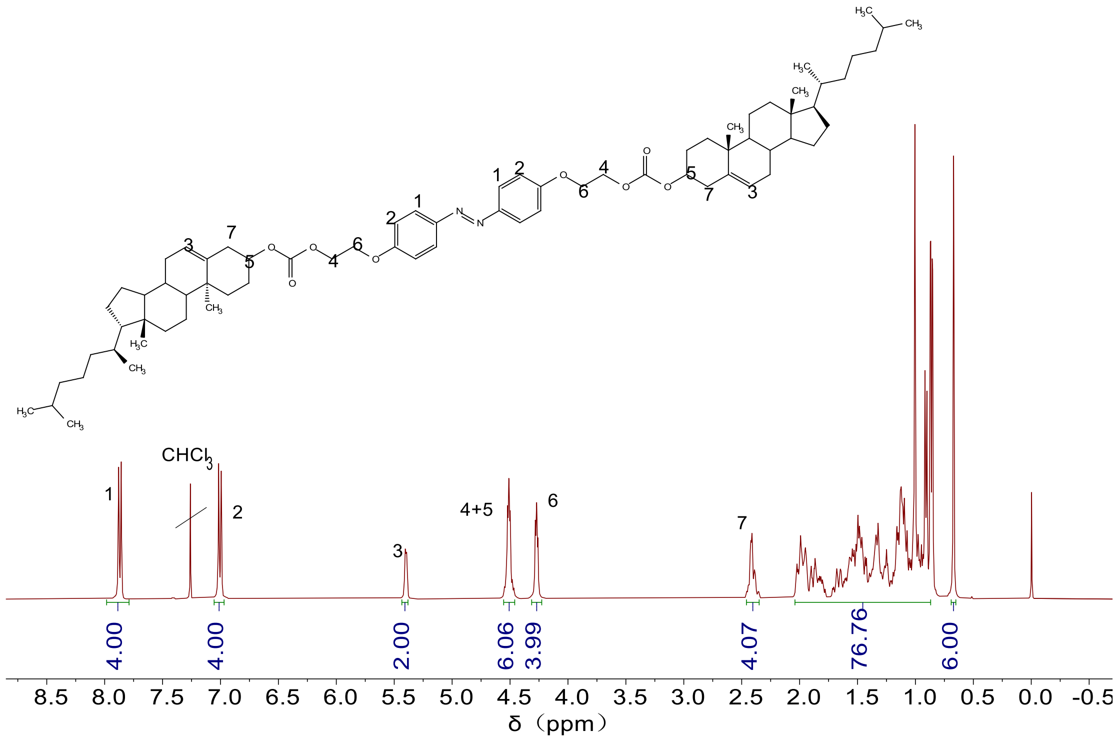

Supplementary Figure 8. ^{1}H NMR spectrum of **Azo-Ch** (400 MHz, $CDCl_3$, 25 °C).

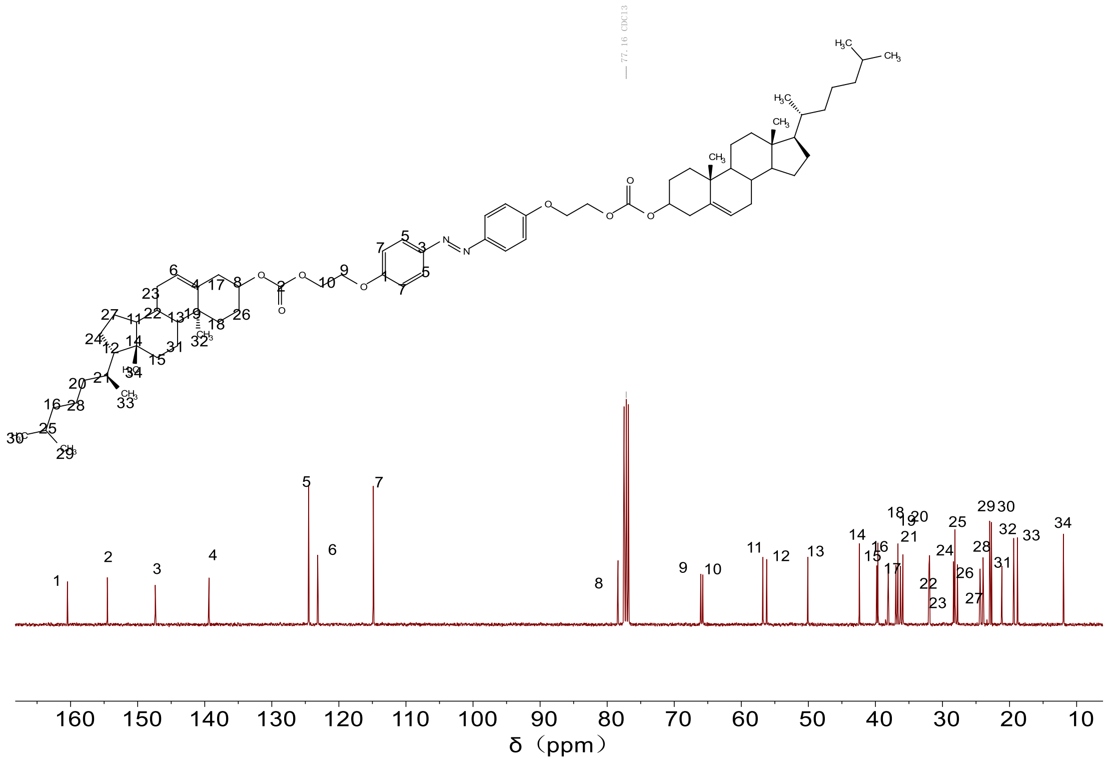

Supplementary Figure 9. ^{13}C NMR spectrum of **Azo-Ch** (100 MHz, $CDCl_3$, 25 °C).

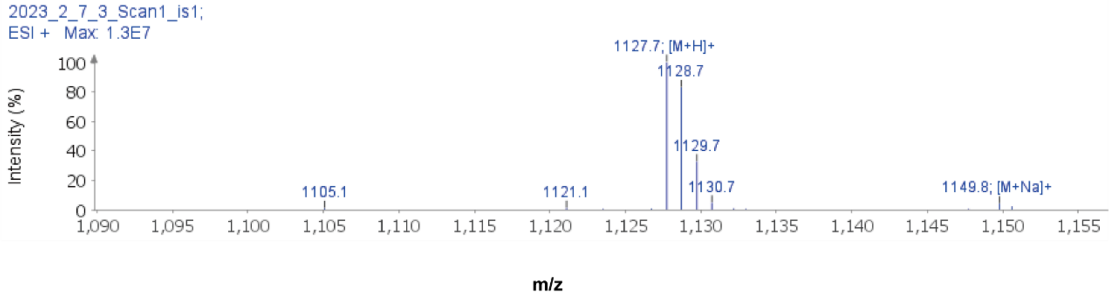

Supplementary Figure 10. MS spectrum of Azo-Ch.

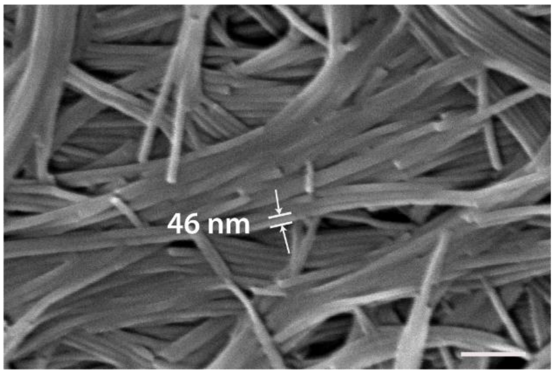

Supplementary Figure 11. SEM images of the Azo-Ch gel. The diameter of a typical fiber is 46 nm. Scale bars: 500 nm.

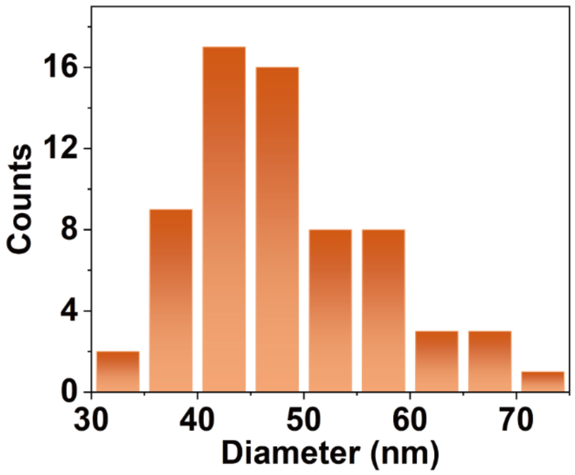

Supplementary Figure 12. The diameter distribution histogram of Azo-Ch fibers measured by SEM. The fibers have a mean diameter of 46 nm.

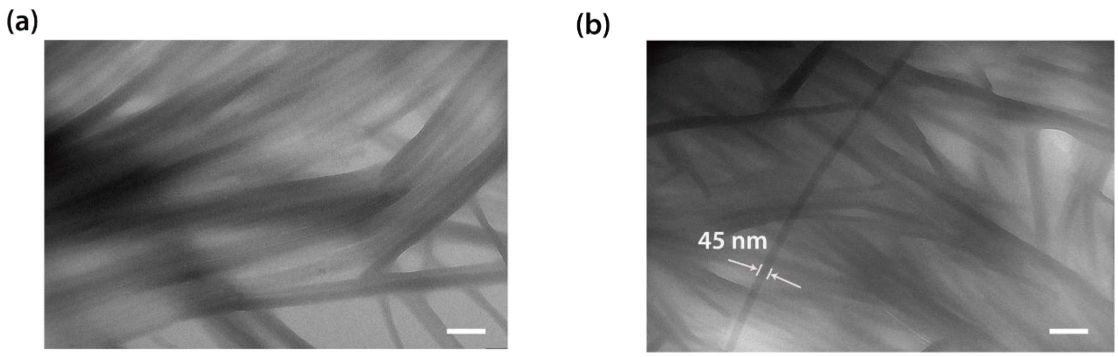

Supplementary Figure 13. TEM images of an Azo-Ch gel measured at two different locations. (a) TEM image of the Azo-Ch gel a location. (b) TEM image of the Azo-Ch gel at another location. The diameter of the fiber is 45 nm. Scale bars: 200 nm.

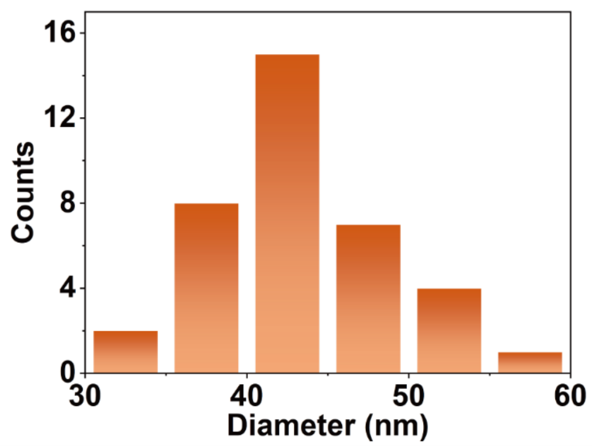

Supplementary Figure 14. The diameter distribution histogram of Azo-Ch fibers measured by TEM. The fibers have a mean diameter of 46 nm.

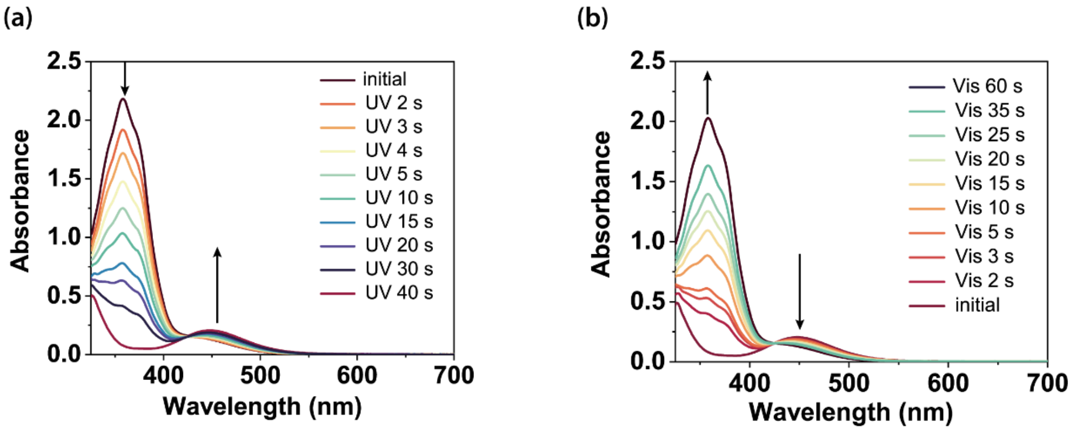

Supplementary Figure 15. UV–vis absorption spectra of the Azo-Ch gel (1% w/v) (a) under irradiation with UV light (365 nm, 12 mW·cm^{-2}) for different time periods and (b) subsequent visible light (530 nm, 15 mW·cm^{-2}) for different time periods.

The *cis* content at the photostationary state was estimated by a method in literature[1,2].

Cis content = $(1\text{-}A/A_{trans})/(1\text{-}\varepsilon_{cis}/\varepsilon_{trans})$ = 92 %

where A is the absorbance of Azo-Ch, A_{trans} is the absorbance of *trans* Azo-Ch, and ε_{cis} and ε_{trans} are the molar absorption coefficients of *cis* and *trans* isomers, respectively, at 359 nm. $\varepsilon_{cis}/\varepsilon_{trans}$ for azobenzene is close to 0.05[2,3].

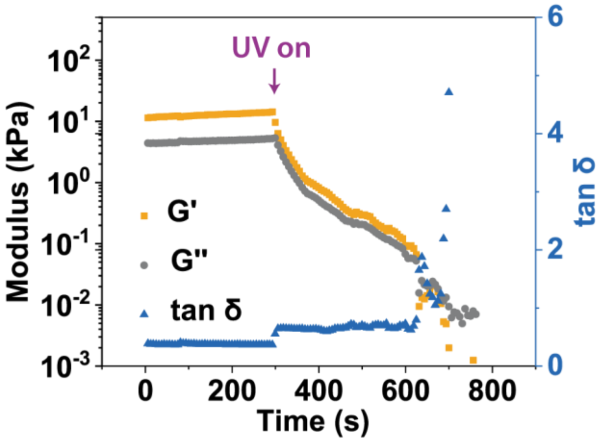

Supplementary Figure 16. The rheology data of the Azo-Ch gel (1 w/v %) upon UV irradiation (365 nm, 30 mW·cm^{-2}) at an oscillation frequency of 10 rad·s^{-1}. Initially, the

storage modulus (G′) of the Azo-Ch gel was larger than the loss modulus (G″), which suggests that Azo-Ch was a gel before irradiation. After UV irradiation, both G′ and G″ decreased. Eventually, G′ was smaller than G″, suggesting that the gel-to-sol transition occurred.

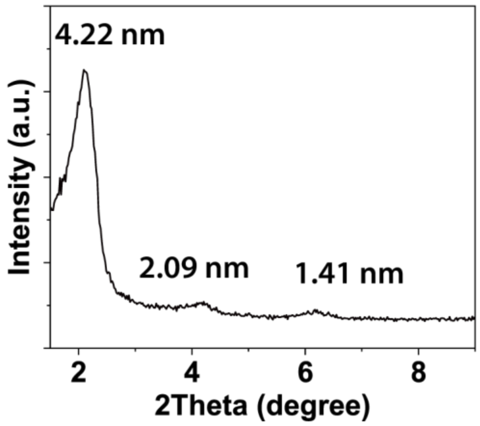

Supplementary Figure 17. XRD pattern of the xerogel of Azo-Ch. The data showed that Azo-Ch formed a lamellar structure in the xerogel.

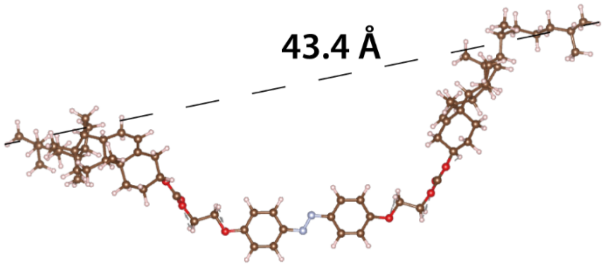

Supplementary Figure 18. 3D structure of Azo-Ch calculated by density functional theory (DFT). Different atoms are labeled with different colors. Light brown: H; dark brown: C; red: O; gray: N.

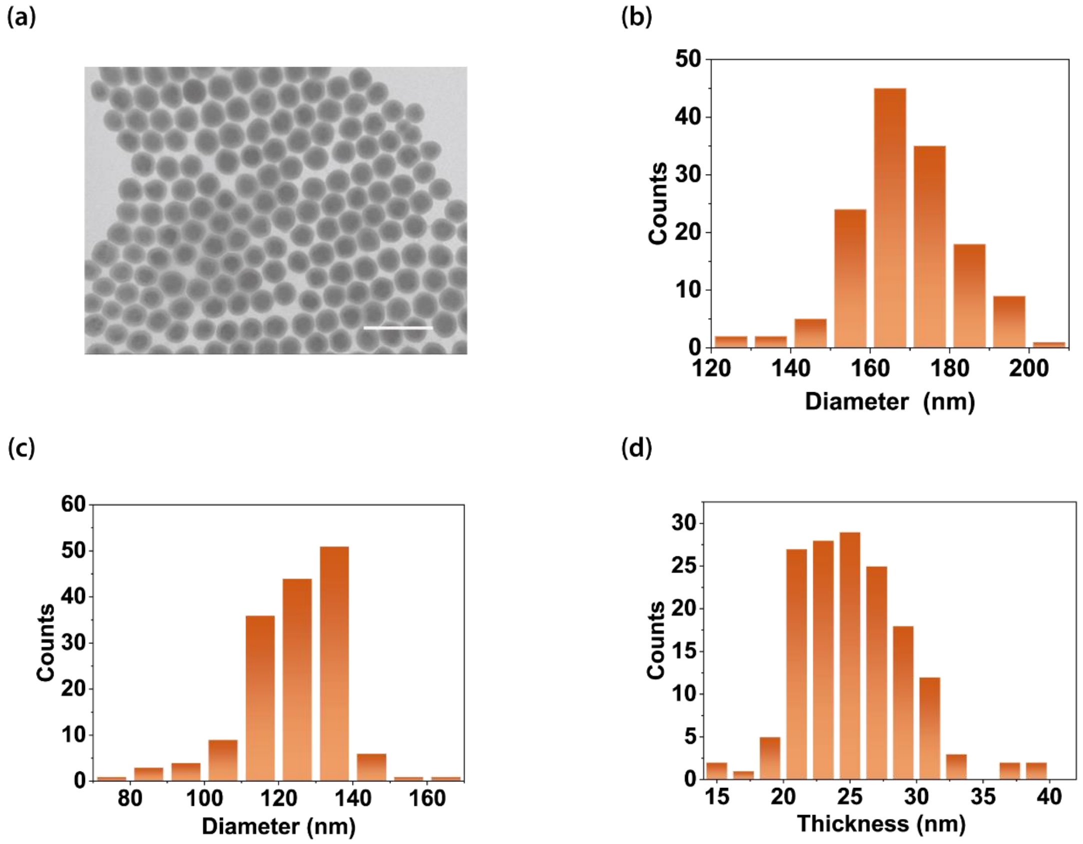

Supplementary Figure 19. TEM image and diameter distribution histograms of Fe_3O_4@SiO_2 nanoparticles. (a) TEM image of Fe_3O_4@SiO_2 nanoparticles. Scale bar: 500 nm. Distribution histograms of the diameters of (b) Fe_3O_4@SiO_2 nanoparticles, (c) Fe_3O_4 core and (d) SiO_2 shell.

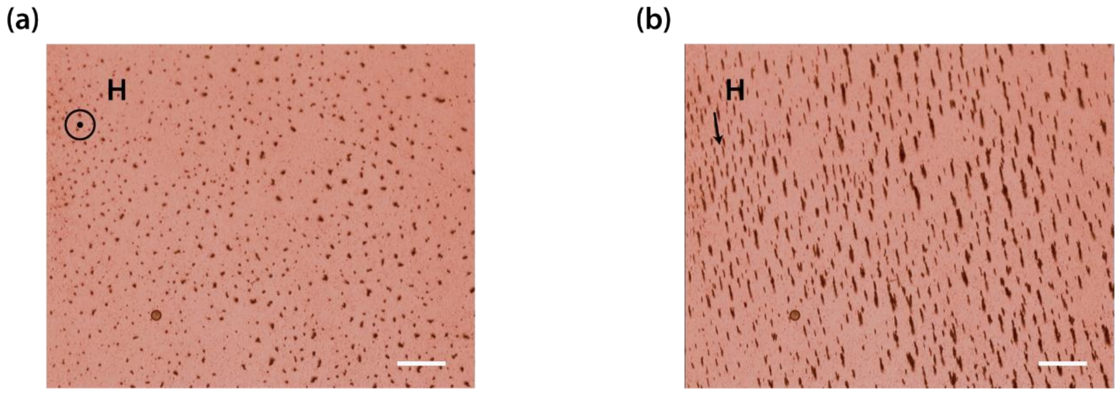

Supplementary Figure 20. Optical microscopy images of Fe_3O_4@SiO_2 nanoparticles under magnetic fields of different directions. H is the vector of the magnetic field. (a) Optical microscopy image with H perpendicular to the plane of the image. (b) Optical microscopy image with H parallel to the plane of the image. The orientation of the Fe_3O_4@SiO_2 nanochains is along the direction of the magnetic field. Scale bars: 200 μm.

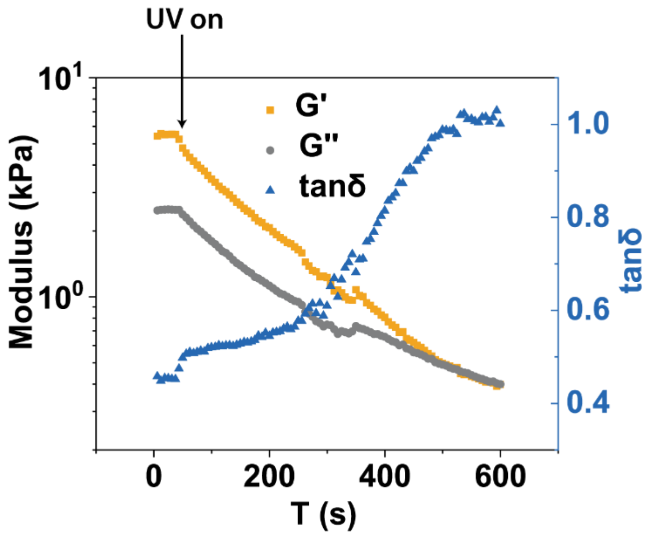

Supplementary Figure 21. Rheology data of the composite gel (8 mg·mL^{-1} Fe_3O_4@SiO_2, 1 w/v % Azo-Ch) upon UV irradiation (365 nm, 30 mW·cm^{-2}) at an oscillation frequency of 10 rad·s^{-1}. Initially, the storage modulus (G′) of the composite

gel was larger than the loss modulus (G″), which suggests that the composite was a gel before irradiation. After UV irradiation, both G′ and G″ decreased. Eventually, G′ was smaller than G″, suggesting that the gel-to-sol transition occurred. The moduli G′ and G″ of the composite gel were comparable to those of the pure Azo-Ch gel in Supplementary Fig. 10.

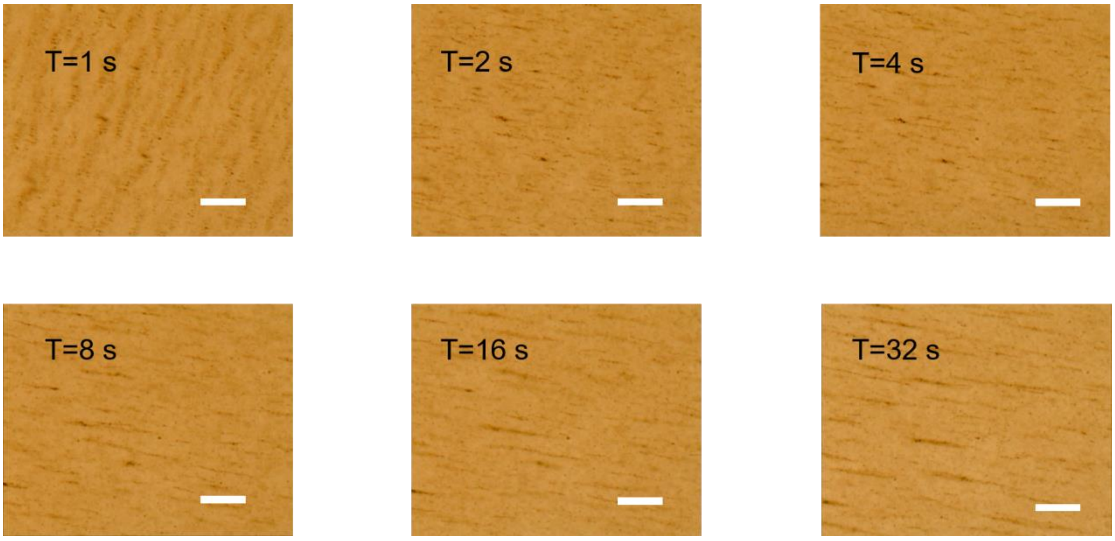

Supplementary Figure 22. Optical microscopy images showing the magnetic-induced formation of nanochains in composite sol (8 mg·mL^{-1} $Fe_3O_4@SiO_2$, 1 w/v % Azo-Ch), which was obtained by irradiating a composite gel with UV light (365 nm, 30 mW·cm^{-2}, 2 min). The length of the $Fe_3O_4@SiO_2$ nanochains increased over time. Scale bars: 20 μm. Magnetic field intensity: 4 mT.

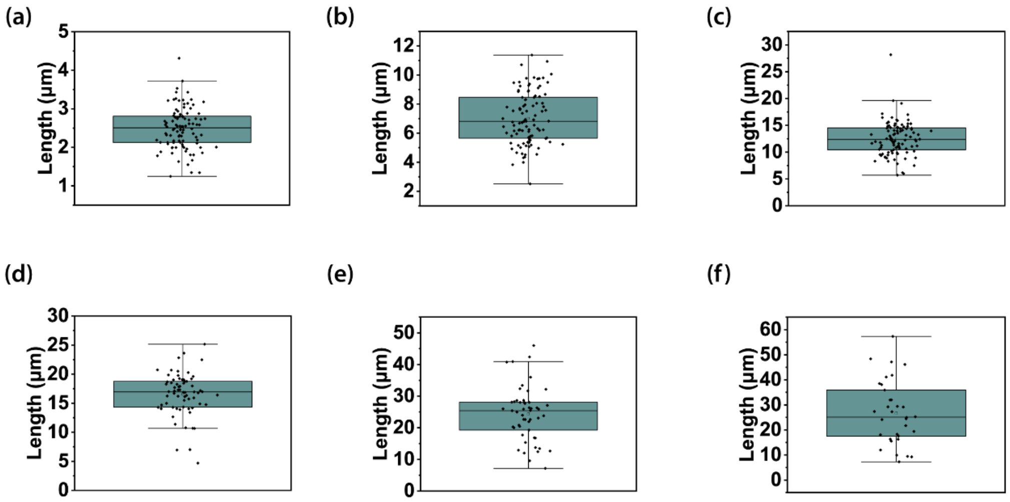

Supplementary Figure 23. Boxplots of Fe_3O_4@SiO_2 nanochains at different time periods: (a) 1 s, (b) 2 s, (c) 4 s, (d) 8 s, (e) 16 s, and (f) 32 s. The box plots indicate median (middle line), 25th and 75th percentile (box), and 1.5× interquartile range (whiskers) as well as outliers (single points). One hundred nanochains were counted for t = 1-16 s. Thirty-four nanochains were counted at t = 32 s because the number of chains decreased. The length of the Fe_3O_4@SiO_2 nanochains increased over time in the sol.

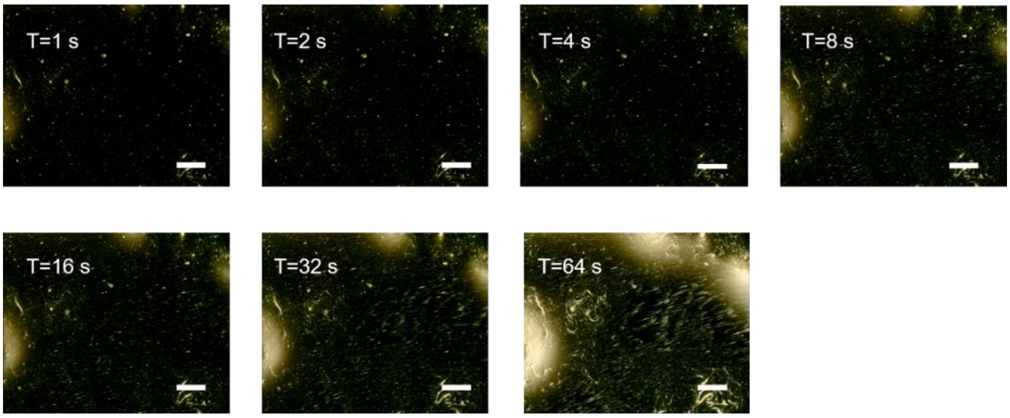

Supplementary Figure 24. Dark-field optical microscopy images showing magetic-induced formation of nanochains in composite gel (8 mg·mL^{-1} Fe_3O_4@SiO_2, 1 w/v % Azo-Ch). The length of the Fe_3O_4@SiO_2 nanochains increased over time in the gel. Scale bars: 200 μm. Magnetic field intensity: 4 mT.

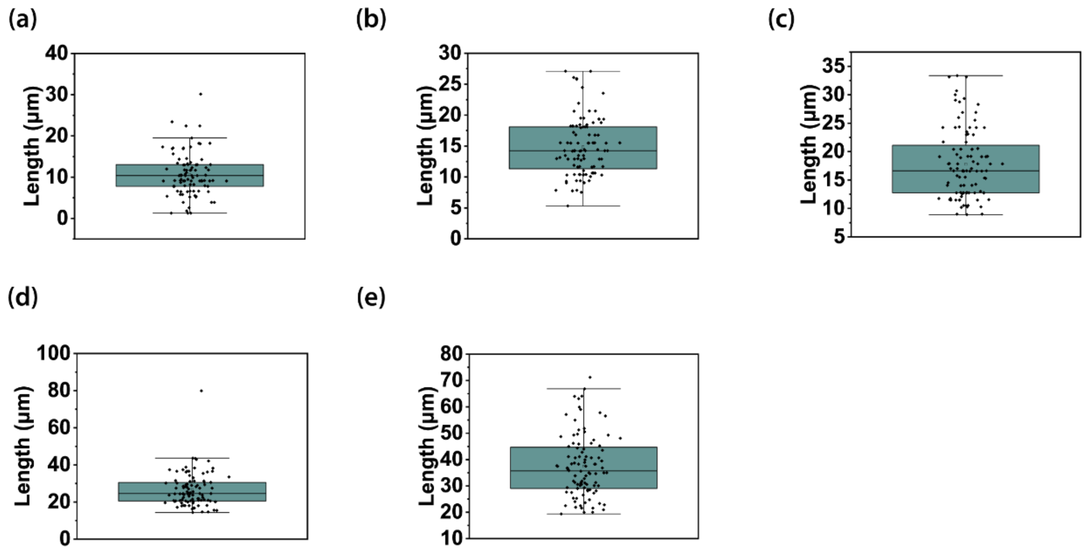

Supplementary Figure 25. Boxplots of Fe_3O_4@SiO_2 chains at different time periods: (a) 2 s, (b) 4 s, (c) 8 s, (d) 16 s, and (e) 32 s. The box plots indicate median (middle line), 25th and 75th percentile (box), and 1.5× interquartile range (whiskers) as well as outliers (single points). 100 nanoparticle chains were counted. The length of the Fe_3O_4@SiO_2 nanochains increased over time.

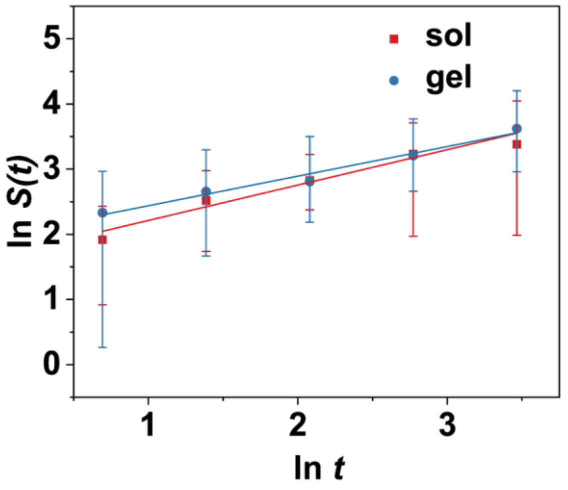

Supplementary Figure 26. Mean chain length $S(t)$ of Fe_3O_4@SiO_2 as a function of time t. Both $S(t)$ and t are plotted using a natural logarithm scale. The straight line

corresponds to asymptotic slopes of $z_1 = 0.54$ (sol) and $z_2 = 0.45$ (gel)[4]. The error bars show the 95% confidence interval.

To understand the formation of nanochains, we studied the assembly of $Fe_3O_4@SiO_2$ nanoparticles in a composite gel or sol under a magnetic field by optical microscopy (Supplementary Fig. 17-20). It is well known that the dynamic scaling law expressed by equation (1) can be applied to the cluster-cluster aggregation process in magnetorheological systems[4-7]:

$$n_s \sim s^{-2} f\left(\frac{s}{t^z}\right), \quad (1)$$

where n_s, s, t, and z are the number of clusters composed of s particles, the number of particles in a cluster, time, and the critical exponent, which characterize the aggregation process. After counting the nanochains and fitting, we found that the value of the asymptotic slope z of the gel was smaller than that of the sol, which suggests that the growth rate of nanoparticles is lower in the gel (Supplementary Table 1).

The motion of each superparamagnetic nanoparticle is expressed by the following Langevin equation (2)[7]:

$$\frac{\partial u}{\partial t} = -\frac{\zeta}{m_p} u + \frac{\mathbf{F}}{m_P} + \mathbf{P} \quad (2)$$

where u, ζ, m_P, and F are the velocity of a nanoparticle, the friction coefficient, the mass of a nanoparticle, and the force acting on a nanoparticle, respectively. We assume that the Stokes law applies to the friction coefficient. P is a random force caused by collisions with solvent molecules. Due to the higher viscosity of the gel system, the friction coefficient is higher than that of the sol, which explains the slower rate for the formation of nanochains.

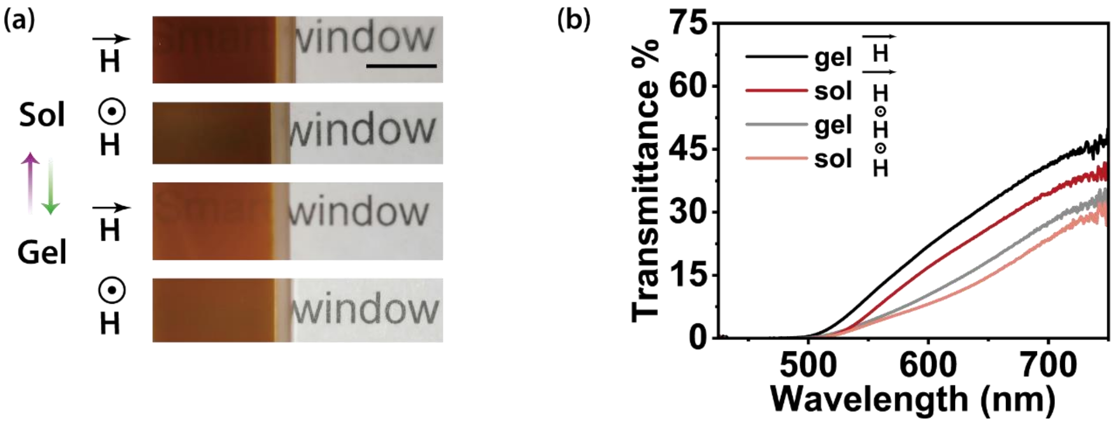

Supplementary Figure 27. (a) Photographs of a smart window controlled by light and a magnetic field (solvent: cyclopentanone; sample thickness: 2 mm); Scale bar: 0.5 cm. (b) Transmission spectra of the smart window controlled by light and a magnetic field.

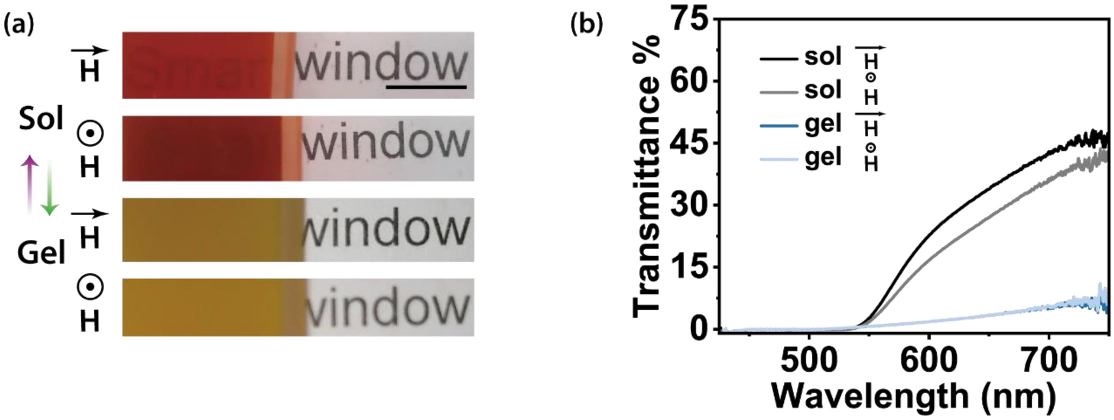

Supplementary Figure 28. (a) Photographs of a smart window controlled by light and a magnetic field (solvent: cyclopentanone/methanol (7/3, v/v); sample thinkness: 10 mm). Scale bar: 0.5 cm. (b) Transmission spectra of the smart window controlled by light and a magnetic field. In comparison, the smart window in Fig. 5 was prepared using an optimized solvent and sample thickness.

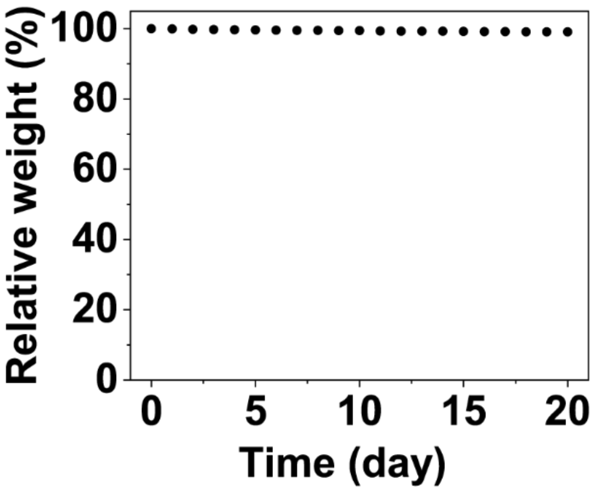

Supplementary Figure 29. Weight change of a smart window for 20 days. The composite gel, which contained cyclopentanone, was sealed between two glasses of the smart window. The smart window showed negligible weight change for 20 days, suggesting that the sealing of the glasses prevented solvent evaporation.

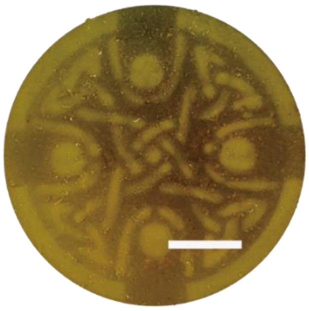

Supplementary Figure 30. A photograph of a complex pattern on a composite gel. Scale bar: 0.5 cm.

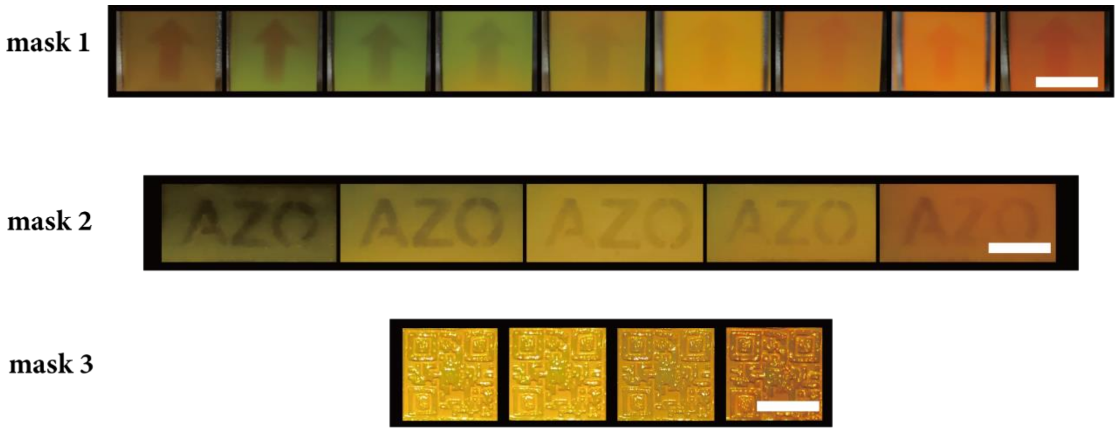

Supplementary Figure 31. Photographs of composite gels with different patterns under magnetic fields (300 mT - 50 mT). Scale bars: 1 cm.

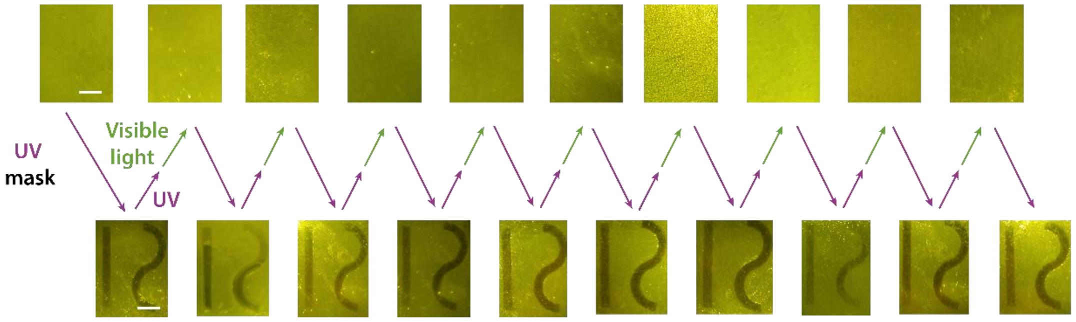

Supplementary Figure 32. Photographs of rewriting a pattern on a composite gel for 20 times using UV (365 nm, 30 mW·cm^{-2}, 30 s) and visible light (530 nm, 40 mW·cm^{-2}, 5 min). Scale bars: 1 cm.

Supplementary Table 1. The length of Fe_3O_4@SiO_2 nanochains in composite gel and sol at different time periods. Mean length was calculated from Supplementary Figs. 22-25.

The length of Fe_3O_4@SiO_2 nanochains		
Time (s)	Mean length of nanochains in sol (μm)	Mean length of nanochains in gel (μm)
2	6.8	10.3
4	12.4	14.2
8	16.9	16.6
16	25.3	24.7
32	29.4	37.4

References

1 Blum, V. et al. Ab initio molecular simulations with numeric atom-centered orbitals. *Comput. Phys. Commun.* **180**, 2175-2196 (2009).

2 Victor, J. G. & Torkelson, J. M. On measuring the distribution of local free volume in glassy-polymers by photochromic and fluorescence techniques. *Macromolecules* **20**, 2241-2250 (1987).

3 Wang, X. Azo polymer colloidal spheres: formation, two-Dimensional array, and photoresponsive properties. In *Smart Light-Responsive Materials*, (Eds. Zhao, Y. & Ikeda, T.) Chapter 5, 177-213 (John Wiley & Sons, Inc., New Jersey, 2009)

4 Vicsek, T. & Family, F. Dynamic scaling for aggregation of clusters. *Phys. Rev. Lett.* **52**, 1669-1672 (1984).

5 Carmen Miguel1, M. & Pastor-Satorras, R. Kinetic growth of field-oriented chains in dipolar colloidal solutions. *Phys. Rev. E* **59**, 826-834 (1999).

6 Melle, S., G. Calderón, O., Rubio, M. A. & Fuller, G. G. Microstructure evolution in magnetorheological suspensions governed by Mason number. *Phys. Rev. E* **68**, 041503 (2003).

7 Tomofumi, U., Hisao M. & Toru M. Cluster-cluster aggregations of superparamagnetic particles in a rotational magnetic field. *Phys. Rev. E* **83**, 061406 (2011).
