# Supplementary material for: Organic‒inorganic semi-interpenetrating networks with orthogonal light- and magnetic-responsiveness for smart photonic gels
Source: Nat Commun. 2023 Feb 22;14:1000. doi: 10.1038/s41467-023-36706-7 (PMC9946997; doi:10.1038/s41467-023-36706-7)
Supplement: Supplementary file 2 — Description of Additional Supplementary Files [file 41467_2023_36706_MOESM2_ESM.pdf]

Description of Additional Supplementary Files

File Name: Supplementary Movie 1
Description: $Fe_3O_4@SiO_2$ nanochains in ethanol controlled by a magnetic field. The nanoparticles moved along the direction of the magnetic field and reformed nanochains that reoriented parallel to the direction of the magnetic field.

File Name: Supplementary Movie 2
Description: Magnetic-responsive structural colors of $Fe_3O_4@SiO_2$ nanoparticles dispersed in ethanol.

File Name: Supplementary Movie 3
Description: Formation of $Fe_3O_4@SiO_2$ nanochains in a composite gel.

File Name: Supplementary Movie 4
Description: Formation of $Fe_3O_4@SiO_2$ nanochains in a composite sol.

File Name: Supplementary Movie 5
Description: A smart window in sol state.

File Name: Supplementary Movie 6
Description: A smart window in gel state.
